# Advanced Hepatitis C Virus Replication PDE Models within a Realistic Intracellular Geometric Environment

**DOI:** 10.3390/ijerph16030513

**Published:** 2019-02-12

**Authors:** Markus M. Knodel, Paul Targett-Adams, Alfio Grillo, Eva Herrmann, Gabriel Wittum

**Affiliations:** 1 Department of Mathematics, Chair of Applied Mathematics 1, Friedrich-Alexander-Universität Erlangen-Nürnberg, Cauerstr. 11, 91058 Erlangen, Germany; 2 Medivir AB, Department of Biology, 141 22 Huddinge, Sweden; Paul.Targett-Adams@medivir.com; 3 Dipartimento di Scienze Matematiche (DISMA) “G.L. Lagrange”, Politecnico di Torino, Corso Duca degli Abruzzi, 24, 10129 Torino (TO), Italy; alfio.grillo@polito.it; 4 Department of Medicine, Institute for Biostatistics and Mathematic Modeling, Goethe Universität Frankfurt, Theodor-Stern-Kai 7, 60590 Frankfurt am Main, Germany; herrmann@med.uni-frankfurt.de; 5 Goethe Center for Scientific Computing (G-CSC), Goethe Universität Frankfurt, Kettenhofweg 139, 60325 Frankfurt am Main, Germany; wittum@gcsc.uni-frankfurt.de; 6 Applied Mathematics and Computational Science, King Abdullah University of Science and Technology (KAUST), 23955-6900 Thuwal, Saudi Arabia

**Keywords:** computational virology, hepatitis C virus (HCV), viral dynamics, within-host viral modeling, mathematical models of viral RNA cycle, population dynamics, 3D spatiotemporal resolved mathematical models, realistic geometries, (surface) partial differential equations, Finite Volumes, massively parallel multigrid solvers

## Abstract

The hepatitis C virus (HCV) RNA replication cycle is a dynamic intracellular process occurring in three-dimensional space (3D), which is difficult both to capture experimentally and to visualize conceptually. HCV-generated replication factories are housed within virus-induced intracellular structures termed membranous webs (MW), which are derived from the Endoplasmatic Reticulum (ER). Recently, we published 3D spatiotemporal resolved diffusion–reaction models of the HCV RNA replication cycle by means of surface partial differential equation (sPDE) descriptions. We distinguished between the basic components of the HCV RNA replication cycle, namely HCV RNA, non-structural viral proteins (NSPs), and a host factor. In particular, we evaluated the sPDE models upon realistic reconstructed intracellular compartments (ER/MW). In this paper, we propose a significant extension of the model based upon two additional parameters: different aggregate states of HCV RNA and NSPs, and population dynamics inspired diffusion and reaction coefficients instead of multilinear ones. The combination of both aspects enables realistic modeling of viral replication at all scales. Specifically, we describe a replication complex state consisting of HCV RNA together with a defined amount of NSPs. As a result of the combination of spatial resolution and different aggregate states, the new model mimics a cis requirement for HCV RNA replication. We used heuristic parameters for our simulations, which were run only on a subsection of the ER. Nevertheless, this was sufficient to allow the fitting of core aspects of virus reproduction, at least qualitatively. Our findings should help stimulate new model approaches and experimental directions for virology.

## 1. Introduction

Viral infections are a major challenge to human health and prosperity. This is not only true for acute outbreaks, e.g., of Ebola infections with high public awareness, but also true for chronic infections that have a major impact on health care management. Infection with the Hepatitis C virus (HCV) [1] causes chronic liver diseases such as liver cirrhosis and liver carcinoma. As chronic infection with HCV affects 2–3 % of the World population, HCV is the main reason for liver transplantations in the Western World [2]. Consequently, HCV receives extensive attention in the scientific research community [1,3,4]. There is no HCV vaccine available but substantial progress has been made in the development of direct acting antiviral agents (DAAs) [5], which specifically target certain virus-encoded gene proteins (e.g., the HCV protease and polymerase). However, there is still considerable room for deeper “mechanistic” insight into the HCV replication cycle. This includes spatiotemporal analysis of HCV RNA translation, replication, and assembly as well as the understanding of the involvement of host factors in these processes [6]. Deeper knowledge of these processes may also have implications for the understanding of fundamental virus–host interactions of related viruses.

Previous mathematical/biophysical models describing HCV replication dynamics at an intracellular level focused on kinetic compartment models (see, e.g., [7,8,9,10,11,12,13,14,15,16,17,18,19]). (Ordinary differential equation (ODE) systems are the basis of kinetic compartment models.) These investigations led to important new insights; however, data and model approaches on single cell HCV RNA kinetics are still limited [20].

Recent experimental work has already generated insight into more detailed spatiotemporal resolved processes of the HCV replication (see, e.g., [3,20,21]). Nevertheless, there is still a limited understanding of the basic processes leading to HCV RNA replication. (Note that viral RNA is sometimes also called “vRNA”). Furthermore, spatial resolution of these processes has received little attention in modeling simulations to date, although strong biological evidence suggests that intracellular spatial dependence is a crucial factor in the process HCV uses to replicate its genome [3,22,23]. Specifically, HCV genome replication occurs in specialized compartments within virus-infected cells called replication complexes (RCs). These RCs are housed within membranous webs (MW), which are built from altered regions of the Endoplasmic Reticulum (ER) [1,3,24,25,26,27].

Formation, function, and trafficking of replication complexes, and their components (viral proteins, HCV RNA, and host factors), is a dynamic process occurring in three dimensions [20,28]. The importance of spatial dynamic patterns of one of the key components of HCV replication and virus assembly (the virus-encoded NS5A protein) is evidenced through the abrogation of HCV replication following application of NS5A inhibitors (NS5aIs) [29,30]. This leads to a spatial redistribution of NS5a [31] and also changes its mobility properties [32,33], which likely contributes to the antiviral effects of the compounds.

In a recent paper [34], we not only gave an overview on spatial virus models and simulations at higher scales, but also introduced fully 3D resolving spatiotemporal models of the HCV RNA replication cycle of HCV. Our starting point was the introduction of spatiotemporal resolved models mimicking the core elements and components of the HCV RNA replication cycle of HCV within a realistic geometric environment at the intracellular level. This HCV RNA replication cycle model consists of a set of surface partial differential equations (sPDEs). However, due to a lack of adequate parameters for the diffusion–reaction equations, the model is more qualitative than quantitative. Therefore, we also built up a modeling framework to estimate the diffusion constant of the HCV NS5A viral protein on the ER surface [35,36,37]. Overall, our previous work on modeling the HCV RNA replication cycle [34] and first spatial parameter estimation [35,36,37] are likely the first attempts in the literature to establish a fully spatiotemporally resolved biophysical description of virus dynamics at an intracellular level.

However, the spatial model described previously [34] focuses on a simplified diffusion-reaction model of the HCV RNA replication cycle. Model parameters include multilinear coefficients leading, e.g., to unrealistic fast depletion of host factors. Furthermore, there is biological evidence that the function of viral (+) strand RNA should be differentiated not only according to the spatial resolution, but also according to the specific role (translation, replication or packaging [20,38]).

Therefore, the scope of this manuscript is to extend our previously published mathematical model of HCV RNA replication dynamics [34]. The new set of sPDEs modeling the HCV RNA cycle overcomes the limitation of multilinear diffusion and reaction coefficients and differentiates between the function of HCV RNA and also between the function of NSPs. Our model was evaluated using the simulation platform UG4 within a Finite Volume method (FV) [39,40]. Using fast multigrid solvers [41], our in silico model was evaluated on fast, massively parallel supercomputers [37]. Despite the more realistic approach and extension of the previous models, most of the parameters are still defined heuristically. Although our simulations were run only on part of the intracellular ER, the new model is now flexible enough to allow adjustments to experimental situations, as in [42,43], and the interplay between in silico and in vitro/in vivo investigations might even suggest new experiments for a deeper understanding of virus dynamics.

## 2. Materials, Methods and Models

### 2.1. Basis of Spatio-Temporal Resolved HCV RNA Replication Cycle Model Development

Experimental approaches in HCV research first focused on studying the HCV RNA replication cycle [1] and subsequently upon viral assembly [6,44]. The basis of our in silico framework is to translate the knowledge from experimental research on the HCV RNA replication cycle into a spatiotemporal resolved mathematical description. Therefore, we start with a realistically reconstructed part of cell environment, namely the ER surface. This enables us to model active and passive transport phenomena undergone by viral components on the ER surface using adequate surface grids.

### 2.2. Hepatoma Cells Used as Basis for Geometry Reconstruction

Huh-7 cells (derived from human hepatoma, non-polarized) were propagated in Dulbecco’s modified Eagle’s medium (DMEM) supplemented with 10% fetal calf serum as described previously [45]. Huh-7 cells were infected with HCV strain JFH-1 and analyzed using confocal microscopy as described by Targett-Adams et al. [22]. We note that these experimental data were generated within a former study.

### 2.3. The Geometric Base for the Model

**Figure 1:**
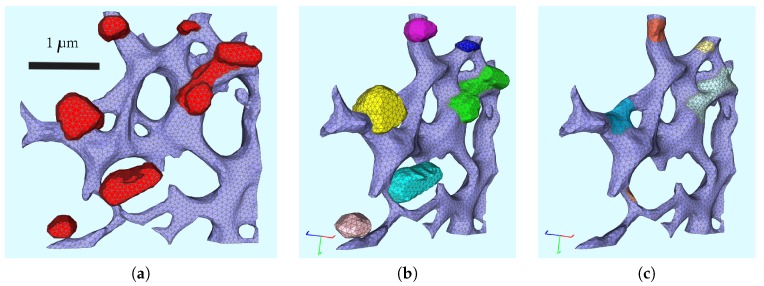
Small subsection of ER, surface grid. All surfaces together form the computation domain 𝒟. (**a**) ER surface and MW regions characteristics: ℰ in blue, and 𝒲 in red. (**b**) Subdomains of the surface model (geometry slightly rotated). Each web region represents its own subdomain. Ribosomes not visible because they are “hidden” behind the web regions. (**c**) Ribosomal regions visible due to undisclosed web regions. Note in (**b**,**c**): blue: ℰ; other colors: single MW regions $W_{i}$ and ribosomal regions $R_{i}$, i=1,2,…,7.

We used fluorescence z-stacks of the cells described directly before (cf. Section 2.2), labeled for the ER surface (calnexin marker) and for the MWs (dsRNA marker). Based on these z-stacks, we used the reconstructed various realistic ER surfaces as described previously [35,36,37] with the aid of NeuRA2 [35,36,46,47]. (For a brief overview over the reconstruction procedure, we refer to Appendix H.) The geometric setup of this study was based on the setup that we described in our former paper [34]. In brief, our spatially resolved model was evaluated on a small subsection of the ER, as shown in Figure 1a.

We denote by D=E∪W the surface obtained by the union of the surface of the ER, ℰ, and the boundaries of the (seven) unconnected MW regions, i.e., $W=\cup_{i=1}^{7}W_{i}$ (see Figure 1, where the web regions $W_{i}$, i=1,1,2,…,7, are marked in red). The ER surface and each $W_{i}$ define a subdomain. For each i=1,2,…,7, the intersection $R_{i}=E\cap W_{i}$ is a subdomain referred to as a ribosomal region. We also set $R=\cup_{i=1}^{7}R_{i}$. The single subdomains depicted with different colors pairwise are shown in Figure 1b. Each of the separated MW regions defines a subdomain, as well as the ER surface, whereas the ribosomal regions are the “root” regions of the ER surface where the MWs are located on top, i.e., the intersection of the MW surface and the ER surface. The single subdomains depicted with different colors pairwise are shown in Figure 1c. The ribosomes are located at the intersection of the ER surface and experimentally defined web regions reflecting the experimental observation of the MW generation close to the NSP creation, $R_{i}=E\cap W_{i}$. Therefore, we define the intersection surface of each MW surface region $W_{i}$ and the ER surface ℰ to be the ribosomal region $R_{i}$.

The rectangular hexahedron enclosing the subsection of the triangular surface mesh is 3.45μm long, 3.36μm high, and 0.87μm deep.

### 2.4. First Simplified sPDE Model

In our former study [34], we introduced a first sPDE model of the HCV RNA cycle upon the afore-mentioned geometric setup. In particular, we assumed that the diffusion process of the viral proteins takes place only on the ER surface and on the surface of the MW regions. The mathematical description of the HCV RNA movement, protein translation, NSP movement, accumulation, and clustering of the NSPs within the geometric MW regions (as reconstructed from the data), HCV RNA copying, and the movement of the new HCV RNA to the ribosomal regions to produce new NSP were summarized by a model with four compartments, which are all regarded as functions of space ($x\in D\subset R^{3}$) and time:

- R(x,t), concentration of HCV RNA
- P(x,t), concentration of the viral polyprotein
- W(x,t), concentration of the web protein
- H(x,t), host factor concentration

We assumed that the “web proteins” are NSPs originating from the cleaved polyprotein and accumulating at the MW regions. The MW regions are defined geometrically by means of the subdomains which arise from the reconstructions of the stained cell data. The accumulation of the “web proteins” (inside the geometrically defined MW regions) forms the entire functional MW. The spatiotemporal evaluation of *R*, *P*, *W*, and *H* is thus modeled by the nonlinear coupled surface PDEs (sPDEs) together with appropriate initial and boundary conditions:

(1a) $$\begin{array}{cc} \partial_{t}R & ={\mathrm{div}}_{T}\left(D_{R}\nabla_{T}R\right)+r_{1}WHR, \end{array}$$

(1b) $$\begin{array}{cc} \partial_{t}P & ={\mathrm{div}}_{T}\left(D_{P}\nabla_{T}P\right)+r_{2}R-r_{3}P, \end{array}$$

(1c) $$\begin{array}{cc} \partial_{t}W & ={\mathrm{div}}_{T}\left(D_{W}\nabla_{T}W\right)+r_{3}P, \end{array}$$

(1d) $$\begin{array}{cc} \partial_{t}H & ={\mathrm{div}}_{T}\left(D_{H}\nabla_{T}H\right)-r_{4}WHR, \end{array}$$

where $D_{R}$, $D_{P}$, $D_{W}$, and $D_{H}$ are the piecewise constant diffusion coefficients of the aforementioned quantities and the reaction rates, $r_{i}\left(x\right)$, i=1,2,3,4, were piecewise constant functions [34].

Here, ${\mathrm{div}}_{T}$ and $\nabla_{T}$ are the tangential divergence and tangential gradient operators, respectively. Note that the diffusion–reaction laws (Equations (1a)–(1d)) are surface differential equations describing the diffusion and reaction processes occurring on the membranes of the ER. The surface diffusion is governed by the Laplace–Beltrami operator, i.e., the projection of the Laplace operator to the tangential space of the two-dimensional ER-hypersurface ℰ, which is embedded into the complete 3D space [48]. (For more detailed explanations and insight into motivations of the mathematical operators div${}_{T}$ and $\nabla_{T}$, we refer to the Appendix G).

In our former paper [34], we also introduced the following notation: Given a (scalar) physical quantity f:D→R and a non-empty set Υ∈D, we write ${\left[f\right]}_{\Upsilon }$ to indicate the restriction of *f* to the set in which it is nonzero. This is done to visualize more clearly when *f* has to be evaluated. Hence, Equations (1a)–(1d) become

(2a) $$\begin{array}{cc} \partial_{t}R & ={\mathrm{div}}_{T}\left(D_{R}\nabla_{T}R\right)+{\left[r_{1}RWH\right]}_{W\cup R} \end{array}$$

(2b) $$\begin{array}{cc} \partial_{t}P & ={\left[{\mathrm{div}}_{T}\left(D_{P}\nabla_{T}P\right)+r_{2}R-r_{3}P\right]}_{R} \end{array}$$

(2c) $$\begin{array}{cc} \partial_{t}W & ={\left[{\mathrm{div}}_{T}\left(D_{W}\nabla_{T}W\right)\right]}_{W\cup R}+{\left[r_{3}P\right]}_{R} \end{array}$$

(2d) $$\begin{array}{cc} \partial_{t}H & ={\mathrm{div}}_{T}\left(D_{H}\nabla_{T}H\right)-{\left[r_{4}RWH\right]}_{W\cup R} \end{array}$$

### 2.5. Extension of the sPDE Model Approach

The sPDE model in Equations (2a)–(2d), has the previously mentioned limitations. As in existing models based on ODEs (see, e.g., [7,8,18]), the reaction term $r_{1}RWH$ is a multilinear function of R,W and *H*. This may be plausible for sufficiently small concentrations R,W and *H* but is now replaced by more realistic terms.

We use concentration dependent reaction terms in Equations (2a)–(2d) to provide more accurate expressions of these quantities. In particular, the new reaction terms tend towards zero for vanishing concentrations and they approach constant values for increasing concentrations. Therefore, the reaction terms $r_{1}WHR$ and $r_{4}WHR$ are replaced by

(3a) $$\begin{array}{c} {\hat{r}}_{1}\left(W,H\right)R={\bar{r}}_{1}\frac{H^{n}}{H^{n}+H_{0}^{n}}\frac{W^{m}}{W^{m}+W_{0}^{m}}R, \end{array}$$

(3b) $$\begin{array}{c} {\hat{r}}_{4}\left(W,H\right)R={\bar{r}}_{4}\frac{H^{n}}{H^{n}+H_{0}^{n}}\frac{W^{m}}{W^{m}+W_{0}^{m}}R \end{array}$$

with constants ${\bar{r}}_{1}$ and ${\bar{r}}_{4}$, the exponents *n* and *m* being positive integers, and the constants $H_{0}$ and $W_{0}$ being reference values of the host factor and web protein concentration, respectively. Then, Equations (2a)–(2d) are replaced by

(4a) $$\begin{array}{cc} \partial_{t}R & ={\mathrm{div}}_{T}\left(D_{R}\nabla_{T}R\right)+{\left[{\hat{r}}_{1}R\right]}_{W}, \end{array}$$

(4b) $$\begin{array}{cc} \partial_{t}P & ={\left[{\mathrm{div}}_{T}\left(D_{P}\nabla_{T}P\right)\right]}_{R}+{\left[r_{2}R-r_{3}P\right]}_{R}, \end{array}$$

(4c) $$\begin{array}{cc} \partial_{t}W & ={\left[{\mathrm{div}}_{T}\left(D_{W}\nabla_{T}W\right)\right]}_{W\cup R}+{\left[r_{3}P\right]}_{R}, \end{array}$$

(4d) $$\begin{array}{cc} \partial_{t}H & ={\mathrm{div}}_{T}\left(D_{H}\nabla_{T}H\right)-{\left[{\hat{r}}_{4}R\right]}_{W}. \end{array}$$

Note that Equations  (3a) and (3b) imply that the reaction terms ${\hat{r}}_{1}\left(H,W\right)$ and ${\hat{r}}_{4}\left(H,W\right)$ are zero when either *H* or *W* is zero. Such conditions hold true, for instance, in the case of the depletion of the host factor described in Equation (4d). Further, these expressions tend to the constant values ${\bar{r}}_{1}$ and ${\bar{r}}_{4}$, respectively, in the limits $H_{0}/H\to 0$ or $W_{0}/W\to 0$. This process is independent of *H*, when the ratio $H_{0}^{n}/H^{n}$ tends towards zero, implying that the depletion rate is independent of *H* when *H* is sufficiently higher than the reference value $H_{0}$.

Additionally, the new model distinguishes between different states of the components, e.g., differentiates between HCV RNA bound to ribosomes and HCV RNA that gets replicated inside of the webs. At the beginning of the HCV RNA replication process, the HCV RNA is assumed to be bound at one ribosomal region $R_{i}$ and may not diffuse outside of it. Moreover, this HCV RNA causes the ribosomes to translate the polyprotein, which is also allowed to diffuse only within the ribosomal region. The concentration of the HCV RNA bound to the ribosomes is denoted by $R_{r}$ and the concentration of the polyprotein is described by *P*. The polyprotein is cleaved into two viral proteins, namely the web protein, with concentration *W*, and another protein (e.g., the non-structural protein 5a, NS5a) with concentration *N*. The web protein is allowed to diffuse within the ribosomal region and the geometrically defined MW subdomain $W_{i}$, and forms the MW within this experimentally defined region. The non-structural protein NS5a, instead, may diffuse freely over the ER surface ℰ and the ribosomal regions R.

We start with modeling the *replication complex* (RC) represented by the concentration *C* as being formed by HCV RNA together with a certain amount of web proteins. This RC may diffuse within the ribosomal region and the MW. The replication process within the RC produces free HCV RNA, $R_{f}$ while consuming also a not further specified host factor *H* during this process. The free HCV RNA may diffuse through the entire domain 𝒟. Besides this, some parts of it will be bound again to the same ribosomal region before escaping. Other parts will diffuse on the ER surface to other ribosomal regions to start the same process that we have observed at the first web: the free HCV RNA is caught at the next ribosomal region to form ribosomal bound HCV RNA, which translates polyproteins, the polyproteins split into the NSPs which create the MW, and so on.

The diffusion coefficients are modeled in a way that they depend not only on the subdomain, but also on the concentration of viral components and host factor. In addition, various processes will only be allowed if enough host factor is still available, i.e., *H* acts as some sort of catalyst for various steps. Hence, the diffusion–reaction equations (Equations (4a)–(4d)) are rewritten as

(5a) $$\begin{array}{cc} \partial_{t}R_{r} & ={\left[{\mathrm{div}}_{T}\left(D_{R_{r}}\nabla_{T}R_{r}\right)\right]}_{R}+{\left[{\tilde{r}}_{1}R_{f}\frac{H^{i}}{H^{i}+F_{1}}-{\tilde{r}}_{2}R_{r}\frac{R_{r}^{p}}{R_{r}^{p}+F_{0}}\frac{W^{j}}{W^{j}+F_{2}}\frac{H^{k}}{H^{k}+F_{3}}\right]}_{R} \end{array}$$

(5b) $$\begin{array}{cc} \partial_{t}P & ={\left[{\mathrm{div}}_{T}\left(D_{P}\nabla_{T}P\right)\right]}_{R}+{\left[{\tilde{r}}_{4}R_{r}\frac{R_{r}^{p}}{R_{r}^{p}+F_{0}}\frac{H^{q}}{H^{q}+F_{6}}\right]}_{R}-{\left[{\tilde{r}}_{5}P\right]}_{R} \end{array}$$

(5c) $$\begin{array}{cc} \partial_{t}N & ={\left[{\mathrm{div}}_{T}\left(D_{N}\nabla_{T}N\right)\right]}_{R\cup E}+{\left[{\tilde{r}}_{5}P\right]}_{R} \end{array}$$

(5d) $$\begin{array}{cc} \partial_{t}W & ={\left[{\mathrm{div}}_{T}\left(D_{W}\nabla_{T}W\right)\right]}_{W\cup R}+{\left[{\tilde{r}}_{5}P-v{\tilde{r}}_{2}R_{r}\frac{R_{r}^{p}}{R_{r}^{p}+F_{0}}\frac{W^{j}}{W^{j}+F_{2}}\frac{H^{k}}{H^{k}+F_{3}}\right]}_{R} \end{array}$$

(5e) $$\begin{array}{cc} \partial_{t}C & ={\left[{\mathrm{div}}_{T}\left(D_{C}\nabla_{T}C\right)\right]}_{W\cup R}+{\left[{\tilde{r}}_{2}R_{r}\frac{R_{r}^{p}}{R_{r}^{p}+F_{0}}\frac{W^{j}}{W^{j}+F_{2}}\frac{H^{k}}{H^{k}+F_{3}}\right]}_{R} \end{array}$$

(5f) $$\begin{array}{cc} \partial_{t}R_{f} & ={\mathrm{div}}_{T}\left(D_{R_{f}}\nabla_{T}R_{f}\right)+{\left[{\tilde{r}}_{6}C\frac{H^{m}}{H^{m}+F_{4}}\frac{W^{n}}{W^{n}+F_{5}}\right]}_{W}-{\left[{\tilde{r}}_{1}R_{f}\frac{H^{i}}{H^{i}+F_{1}}\right]}_{R} \end{array}$$

(5g) $$\begin{array}{cc} \partial_{t}H & ={\mathrm{div}}_{T}\left(D_{H}\nabla_{T}H\right)-{\left[{\tilde{r}}_{3}C\frac{H^{m}}{H^{m}+F_{4}}\frac{W^{n}}{W^{n}+F_{5}}\right]}_{W} \end{array}$$

The parameters and coefficients of this system have the following characteristics: The exponents *i*, *j*, *k*, *m*, *n*, *p*, and *q* are positive integers, ${\tilde{r}}_{1},\ldots ,{\tilde{r}}_{6}$ are real constants with physical units $\left[s^{-1}\right]$ and represent the characteristic values of the reaction terms in Equations (5a)–(5g). The parameter v>1 is a real constant introduced to highlight the fact that the adsorption rate of the web-proteins must be larger then that pertaining to the ribosomal HCV RNA (in fact, v≫1). The parameters $F_{0},\ldots ,F_{6}$ are all positive and therefore cause that the reaction rates have constant asymptotic values for increasing values of the species concentration. The diffusion coefficients $D_{R_{r}}$, $D_{P}$, $D_{N}$, and $D_{W}$ are taken as constants, whereas $D_{C}$, $D_{R_{f}}$, and $D_{H}$ are concentration dependent and are given by

(6a) $$\begin{array}{c} D_{C}:={\hat{D}}_{C}\left(W\right)=D_{C}^{\left(0\right)}\frac{W}{W+F_{7}}, \end{array}$$

(6b) $$\begin{array}{c} D_{R_{f}}:={\hat{D}}_{R_{f}}\left(W,N\right)=D_{R_{f}}^{\left(0\right)}\left[F\frac{N}{N+F_{8}}+\frac{W}{W+F_{9}}\right], \end{array}$$

(6c) $$\begin{array}{c} D_{H}:={\hat{D}}_{H}\left(W,N\right)=D_{H}^{\left(0\right)}\left[G\frac{N}{N+F_{10}}+\frac{W}{W+F_{11}}\right]. \end{array}$$

In Equations (6a)–(6c), $D_{C}^{\left(0\right)}$, $D_{R_{f}}^{\left(0\right)}$, and $D_{H}^{\left(0\right)}$ are constant diffusion coefficients, and F and G are model parameters tuning the influence of the terms $N/(N+F_{8})$ and $N/(N+F_{10})$ on $D_{R_{f}}$ and $D_{H}$, respectively, and $F_{7},\ldots ,F_{11}$ have an equivalent meaning, similar to $F_{0},\ldots ,F_{6}$.

Equations (5a)–(5g) are solved by enforcing Neumann zero boundary condition on ∂D and employing the initial conditions

(7a) $$\begin{array}{cc} R_{r}\left(x,t_{0}\right) & =\left\{\begin{array}{cc} R_{r0}, & \forall x\in R_{i_{⋆}},\mathrm{with}i_{⋆}\in \left\{1,\ldots ,7\right\}\;\left(\mathrm{we}\;\mathrm{choose}\;i_{⋆}=2\right),\\ 0, & \forall x\in D\backslash R_{i_{⋆}}, \end{array}\right. \end{array}$$

(7b) $$\begin{array}{cc} H(x,t_{0}) & =H_{0},\;\forall \;x\in D, \end{array}$$

(7c) $$\begin{array}{cc} \left.\begin{array}{c} P(x,t_{0})\\ W(x,t_{0})\\ N(x,t_{0})\\ C(x,t_{0})\\ R_{f}\left(x,t_{0}\right) \end{array}\right\} & =0,\;\forall \;x\in D. \end{array}$$

Various NSPs are involved in the HCV RNA replication cycle (NS2, NS3, NS4a, NS4b, NS5a, and NS5b). However, among all these, NS5a (described by the concentration *N*) plays a crucial role [11,21,30,31,32,33,49,50,51,52,53], since it seems to be involved in all steps of the HCV RNA replication. Hence, our component *N* refers to the freely diffusing part of NS5a. However, the web protein refers to that fraction of NSPs which accumulates to create double membrane vesicles (DMV) and thus the MW. Therefore, the “web protein” is considered to be a combination of all of the NSPs within our present modeling approach still.

Finally, we introduce the abbreviations:

(8) $$G_{C}={\tilde{r}}_{2}R_{r}\frac{R_{r}^{p}}{R_{r}^{p}+F_{0}}\frac{W^{j}}{W^{j}+F_{2}}\frac{H^{k}}{H^{k}+F_{3}}$$

and

(9) $$\begin{array}{ccc} A_{C}^{W} & = & \frac{W^{j}}{W^{j}+F_{2}} \end{array}$$

(10) $$\begin{array}{ccc} A_{C}^{R} & = & \frac{R_{r}^{p}}{R_{r}^{p}+F_{0}} \end{array}$$

(11) $$\begin{array}{ccc} A_{C}^{H} & = & \frac{H^{k}}{H^{k}+F_{3}} \end{array}$$

with j,k,p∈N. These abbreviations simplify a detailed analysis of the new form of replication complex creation as modeled by means of Equation (5e) (see the Appendix C.3).

Note: We use the same factor $A_{C}^{R}$ for polyprotein translation and replication complex construction, since we assume that both processes require a similar amount of ribosomal bound HCV RNA.

### 2.6. Well-Posedness of the Model

To ensure the well-posedness of the problem given by Equations (5a)–(5g) and the considered set of initial and boundary conditions, one should prove that the problem admits a unique solution, and that such solution depends on the input data in a continuous way. However, since the non-linearity of Equations (5a)–(5g) makes it difficult to construct a rigorous proof, one can look at the well-posedness of a linearized version of the problem. To this end, we initially notice that each equation of the system in Equations (5a)–(5g) describes a nonlinear diffusion–reaction process, with the non-linearity featuring both in the reaction terms and in the diffusion coefficients of Equations (5a)–(5g). In addition, we notice that the subsystems in Equations (5a)–(5d) and Equations (5e)–(5g) exhibit a remarkable difference: While the diffusion coefficients of Equations (5a)–(5d) are strictly positive constants, those of Equations (5e)–(5g) are functions of *W* and *N*. Such functions, in turn, are positive only when *W* and *N* are different from zero, and are equal to zero otherwise. Hence, a characterization of the well-posedness of Equations (5a)–(5g) should be done by distinguishing between the case in which *W* and *N* are nonzero from the cases in which *W* and *N* are zero, or at least one of them is zero. For the sake of brevity, we consider here only the first two cases.

When *W* and *N* are both nonzero, all the diffusion coefficients of the system in Equations (5e)–(5g) are strictly positive. In this situation, the problem complies with the hypotheses of the Lax–Milgram Theorem. Consequently, each of the bilinear forms arising from the weak formulation of the linearized problem turns out to be continuous and coercive (please see [54,55] for details), and the solution is unique and stable in the sense of the Lax–Milgram Theorem [54].

When *W* and *N* are both zero, the diffusion coefficients $D_{C}$, $D_{R_{f}}$, and $D_{H}$ vanish identically. Equations (5e)–(5g) become ordinary differential equations, while Equations (5a)–(5d) are partial differential equations, all characterized by positive definite diffusion coefficients. The problem, in this case, is well-posed. In particular, the initial and boundary conditions can be chosen in such a way that the only solution to Equations (5a)–(5g) is the null one, i.e., $R_{r}=P=N=W=C=R_{f}=H=0$.

Since an explicit mathematically well-founded proof of existence and uniqueness of at least weak solutions of the entire general nonlinearized system in Equations (5a)–(5g) is beyond the scope of the present study, we studied the behavior of the simulation data under grid refinement; when we iteratively refined our computational grid to increase resolution, we saw the data remained consistent. The grid refinement studies are shown in the Appendix F. The demonstration of grid convergency may be considered as a strong hint that the system we use is mathematically well-posed, even though it does not replace a formally rigorous proof. Indeed, we intend to elaborate an extended rigerous, mathematically strict proof as part of a future project.

From the biological point of view, the “well-posedness” of Equations (5a)–(5g) is related to their capability of describing the virus dynamics in a physically sound way. This requires one to express the diffusion coefficients and the reaction terms through constitutive laws determined experimentally. In the absence of more precise experimental information on this subject, we fulfill this task by hypothesizing that the diffusion coefficients and the reaction terms follow the theory of population dynamics. In doing this, we introduce several reference parameters and exponents with the purpose of making our model as flexible as possible.

### 2.7. Technical Details of the Solution of the sPDEs

The set of Equations (5a)–(5g), along with the boundary conditions and the initial conditions  in Equations (7a)–(7c), was solved numerically by adopting the Finite Volume method [36] and enforcing a geometric multigrid (GMG) cycle involving up to four grid levels. The problem consists of 39,515 degrees of freedom (DoFs) on the coarse grid and about 10 millions of DoFs on the finest level we use, which is grid refinement level 4.

The node number at base level and the number of the DoFs of the sPDE evaluations at all levels used for evaluation are shown in Table 1. Hence, the simulations presented afterwards were performed within a four-fold spatial refinement GMG environment, and the results were compared to coarser levels, inclusively the base level without refinement.

The numerical simulations were performed with the aid of the software toolbox UG4 [40,41].

### 2.8. Parameter Set Used for the Numerical Evaluation of the sPDE System

The basic parameters we used for demonstration purposes of our new model, i.e., of Equations (5a)–(5g), are reported in Table 2 and Table 3.

We emphasize that our program code is not limited to these set of parameters; we only used it for demonstration purposes.

Finally, we note that the surface diffusion coefficient of the NSPs (i.e, web accumulating protein in the geometric MW regions and free NS5a on the ER surface) are chosen differently. We assume that free NS5A diffuses more quickly than NSPs inside the MWs [35].

### 2.9. Integrals of Concentrations over Subdomains

To evaluate the simulations, we computed the integrals of the concentrations over distinct subdomains. For example, to evaluate the integral of the concentration of ribosomal bound RNA over the ribosomal region number 2, such an integral would be denoted by

(12) $$I_{R_{r}}\left(R_{2}\right):=\int_{R_{2}}R_{r}\;d\sigma .$$

More generally, we employ the notation

(13) $$I_{Q}\left(S\right):=\int_{S}Q\;d\sigma .$$

where *Q* is the generic concentration, and S is the generic surface over which the integration is performed.

### 2.10. 1D Trajectories on the ER Surface for Concentration Profiles Evaluation

**Figure 2:**
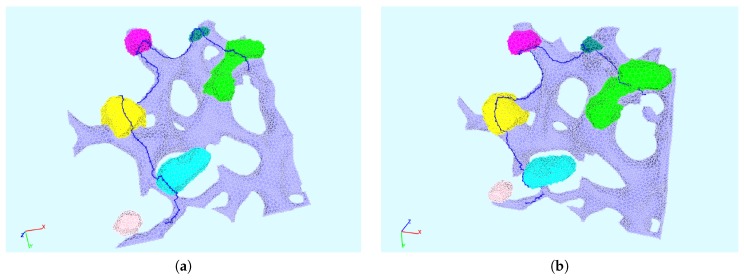
Evaluation of the concentrations along the shown pathways (two perspectives of the path: path indicated by lines in blue, starting from the upper right direction anti-clockwise). (**a**,**b**) show the geometry/the path from slightly changed perspectives.

**Figure 3:**
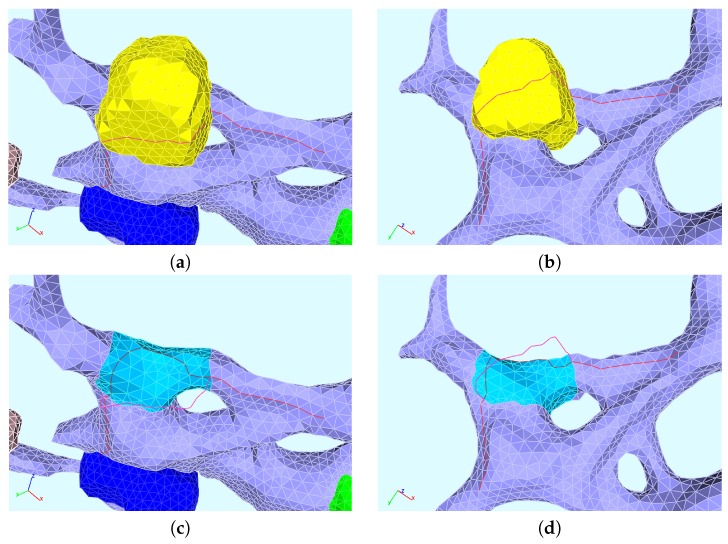
Evaluation of the concentrations along the shown pathways (four perspectives of the path: path on ER and web surface, path on the ribosomes, with and without the web visible). Path from right to left. The sub paths are the four red lines, which start/end where another subdomain begins (besides the external beginnings on the ER, left and right). (**a**) path on ER and web surface, web visible, (**b**) path on ER and web surface, web visbile, slightly changed perspective, (**c**) path on ER and ribosomes, web invisible, (**d**) path on ER and ribosomes, slightly changed perspective.

We evaluated the profiles of concentrations upon 1D pathways for special cases. Therefore, we chose two types of paths:

- One long path, which is located along the ER and crosses various web regions. On this path, we evaluated the concentrations of free RNA and host factor to enable a global overview. The path is depicted in Figure 2.
- The shorter path resolves the processes around and within one single web region. The path is shown in Figure 3. This path decomposes into four subsections, which we call “sub paths” in the forthcoming:
  – The “right” path is the direction from where the free RNA originates. This means that this sub path is located in the direction of a web that was hijacked before by the virus particles. On this path, we show the concentrations of free RNA, host factor, and NS5a.
  – The “upper” path is located on the outer surface of the web, and we evaluated the concentrations of free RNA, host factor, web protein, and replication complex.
  – The “lower” path is located at the ribosomal region, which belongs to the web region. On this path, we evaluated free RNA, ribosomal bound RNA, replication complex, polyprotein, web protein, NS5a, and host factor.
  – The “left” path is that sub-path where there is nearly no free RNA located when the corruption of the web region under consideration starts, since it is on the “opposite” side of the “influx” direction of the free RNA. Therefore, the major part of the free RNA synthesized at the web region under consideration will diffuse away on this path in order to hijack the next web region. As in the case of the “right” path, we show the concentrations of free RNA, host factor and NS5a.

**Figure 4:**
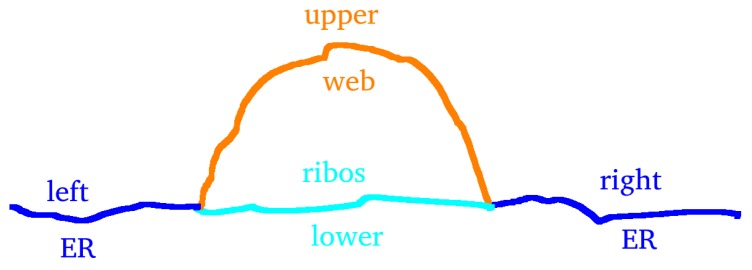
Schematic description of the four different sub paths around the web, as shown in Figure 3 (cf. Section 2.10). The ordering of the sub paths shown in this figure corresponds to the perspective as shown in the realistic geometry screenshot in Figure 3b,d.

We mention that, on each of these sub paths, only the depicted concentrations may have values different from zero due to the given model structure. A schematic presentation of the sub paths is shown in Figure 4.

The directions of evaluation were anti-clockwise for both types of paths. In the case of the short path, the concentration profiles were plotted as indicated by the location (although the path lengths used as *x*-axis may cause confusion due to their direction), while the results of the long path were plotted directly upon the path length.

### 2.11. General Notes On Scaling Within Figures

Note that in the case of all figures of this paper, the factors given in the legends of certain concentrations have to be understood as multiplication factors: For example, if we write host[10], this means that the displayed value of the host factor has to be multiplied with a factor of 10 in order to correspond to the value as given by the simulation (value 200 in the figure indicates value “host” 20 in reality/in simulation). Or, if we write web[10^−4^], this means that 1 “web” in the figure corresponds to 10,000 web proteins in reality/in simulation. The scaling is performed to allow for visualization of the values of different components within one figure.

## 3. Results

### 3.1. Simulation of the Population Dynamics Inspired sPDE Model

We consider simulations of the basic set of Equations (5a)–(5g). The diffusion coefficients are defined in Equations (6a)–(6c). The initial conditions are given by Equations (7a)–(7c). The test parameters are those reported in Table 2 and Table 3.

**Figure 5:**
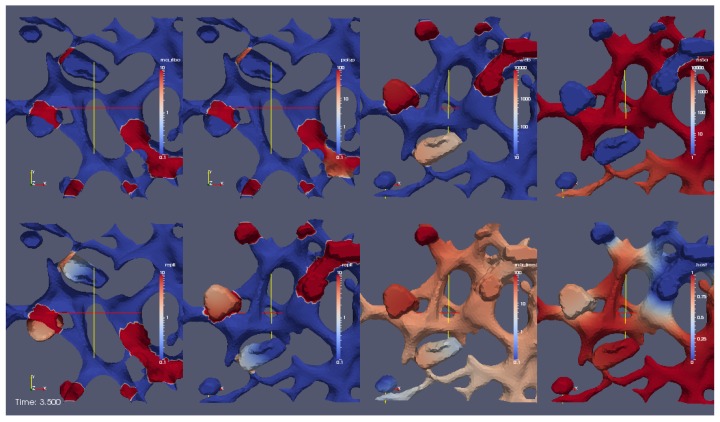
Screenshot of the simulation of the improved surface model as visualized in movie “Video S1—Population Dynamics Inspired sPDE Model Static View” (description cf. Section 3.1, movie attached as Supplemental S1). Each sector depicts one concentration. In part, different perspectives are used. Upper row, from left to right: Ribosomal-bound HCV RNA, polyprotein, web protein, and NS5a. Lower row, also left to right: Replication complex (ER disclosed by means of a cutting plane), replication complex (ER undisclosed), free HCV RNA, and host factor. For detailed description, see Section 3.1.1. In brief, the movie shows how ribosomal-bound HCV RNA translates polyprotein. The polyprotein splits into web protein and NS5a. The web protein accumulates at the geometrically defined web region to form the MW. NS5a diffuses away at the ER surface. One formerly ribosomal-bound HCV RNA and several web proteins cluster together to form the RC, which diffuses into the MW, where it polymerizes free HCV RNA. The newly synthesized free HCV RNA in part diffuses away and in other parts becomes bound again at the ribosomes on-site. When free HCV RNA reaches the next ribosomal region, the cycle starts there again. Step by step, the cell is filled with HCV RNA and viral proteins. The host factor is consumed where (free) HCV RNA gets polymerized by the RC.

A movie in which the whole simulation can be visualized is attached as Supplemental S1, “Video S1—Population Dynamics Inspired sPDE Model Static View” (cf. Section 3.1.1 for an extended description, movie cf. Supplemental S1). A frame (screenshot) of this simulation movie is shown in Figure 5, where each image represents the spatial distribution of the concentration of one of species accounted for in the model. (The time scale of the screenshot at time t=3.5s is qualitative due to the qualitative character of the model parameters, i.e., also of the time scale.) The images in the first row describe, from left to right, the distributions of the ribosomal bound HCV RNA, $R_{r}$, polyprotein, *P*, web protein, *W*, and NS5A, *N*. The first two images of the second row, instead, describe the distribution of the replication complexes, *C*, viewed by two different perspectives. Moreover, the third and fourth image of the second row report the concentration of the free HCV RNA, $R_{f}$, and of the host factor, *H*, respectively. In detail, we observe processes which we describe in the forthcoming section.

#### 3.1.1. Video S1—Detailed Description

In the upper left sector, we observe the evolution of the concentration of ribosomal bound HCV RNA ($R_{r}$). This sector deploys a part of the computational domain. This part was generated by a cut of the computational domain by means of a cut plane. This procedure allows for the visualization of the initial concentration of ribosomal bound HCV RNA $R_{r}\left(x,0\right)$. Without using a cut plane, it would be impossible to observe the evolution of the concentration of ribosomal bound HCV RNA $R_{r}$, since it can only be different from zero at the ribosomes. We recall that we model the ribosomes such that they are located at those spatial points in which the ER surface intersects with the MW regions. These ribosomal regions are hidden unless the domain is disclosed by means of a cutting plane. Hence, we observe the concentration of ribosomal bound HCV RNA from a backward perspective, upon the computational domain that is disclosed by means of the cutting plane. This effectively allows for observing the ribosomal region (where the first initial ribosomal bound HCV RNA concentration is located) from inside. At the beginning, HCV RNA is attached to ribosomes and may not move away. The ribosomal bound HCV RNA may only translate viral polyprotein.

Indeed, in the second upper sector from the left of the movie, we observe that the ribosomal bound HCV RNA $R_{r}$ translates the polyprotein *P*. The perspective of this sector is the same as before for the ribosomal bound HCV RNA. The polyprotein cleaves into web (accumulating) protein and another NSP that may move freely on the ER surface, which we called NS5A. (We recall that the NSP has properties reminiscent of NS5A but could be a different NSP or a combination of some NSPs, without restriction of the generality.) We observe the concentrations of the cleaved web protein and the cleaved NS5A within the two sectors of the upper right of the simulation movie “S1 Video—Population Dynamics Inspired sPDE Model Static View”. The NSPs anchor to the ER surface. The web protein accumulates at the geometric MW regions. NS5A diffuses away on the ER surface. In detail, the second sector from the upper right deploys the evolution of the web protein, i.e., *W*, while the sector on the upper right deploys the evolution of NS5A, i.e., *N*. Note that the perspectives of *W* and *N* are different from those before, i.e., we consider the scene for the concentration dynamics of *W* and *P* from another viewpoint than those those of $R_{r}$ and *P*. The web protein and NS5A are shown from the front perspective, and without a cut plane. We observe that the web protein accumulates at the experimentally based geometric MW regions. Hence, the web protein accumulation causes the MW regions to grow and to get functional and active. Our free NS5A does not move into the webs; moreover, it diffuses away at the ER surface.

Let us consider the lower sectors of the simulation movie: The first two sectors on the lower left deploy the replication complex (RC, whose concentration is denoted by *C*), which arises once enough web protein is available, or, in other words, the RC is constructed once the MW gets dense. The evolution of the concentration of the replication complex is visualized within the first two sectors of the lower left of the simulation movie “S1 Video—Population Dynamics Inspired sPDE Model Static View”. For a sufficient amount of web protein (visible in the second sector from the upper right), one observes how the RC arises (visible in the sectors at the lower left). In detail, once a substantial amount of web proteins has filled the geometrically defined web regions, one ribosomal bound HCV RNA $R_{r}$ dissolutes from the ribosomes and “picks up” a defined amount of web proteins *W* to form one RC *C*. This behavior is due to the fact that the reaction term in Equation (5e) allows for the coupling of web proteins and ribosomal bound HCV RNA to form the RC only when the web protein concentration reaches a certain level. The number of web proteins involved in the RC formation is greater than the number of involved HCV RNAs (Equation (5a)), due to the factor v≫1 in Equation (5d).The number of RCs per MW region is not restricted to one but depends on the local concentrations of available species. This combination of ribosomal bound HCV RNA and web proteins is a bound state, and forms the RC. The relation HCV RNA/web protein number remains practically constant within each RC. This is an important new feature of our model structure. The relation of HCV RNA and web proteins within an RC is realized by means of a parameter of our model. We also assume that, within in vivo/in vitro cell experiments, the number of NSPs that are involved in the replication of one HCV RNA is more or less always similar. Therefore, our description is close to biological reality. (This feature cannot be realized by means of multilinear reaction coefficient models. For a detailed discussion of our new and precise mathematical form referring to the RC creation, we refer to the Appendix C.3).

The simulation shows that the replication complex diffuses into the MW region (i.e., the subdomain that is based on the reconstruction of the stained cell data). We use two different sectors for RC observation in order to be able to observe the RC from two different perspectives. The first sector at the lower left visualizes the RC concentration by means of the cut plane from the perspective of behind. This viewpoint indicates that the ER and the MW regions are opened, and we also observe the ribosomal regions. The second (lower) sector from the left deploys the RC concentration from the front and without using a cut plane. Thus, we see the webs directly from the front, the ribosomal regions being hidden.

The RC may only diffuse within the web regions and only to such regions where the web is already dense, i.e., the RC may only diffuse into those regions of the geometrically defined web regions where already enough web proteins have accumulated. Once the RC diffuses into the MW region, it polymerizes new free HCV RNA, $R_{f}$, as required by Equation (5f).

At the third upper sector from the left (equivalently the second sector from the right), we observe the free HCV RNA concentration. Indeed, with increasing RC concentration, free HCV RNA is produced. In this movie, a front view of the web regions and of the ER is provided. While the new free HCV RNA is polymerized, the host factor *H* is consumed, as shown by Equation (5g). The host factor consumption is practically constant per newly synthesized HCV RNA. (In addition, this property, even though in principle natural, only arises due to population dynamics and is not the current state of the art). The host factor depletion is visualized within the lower right picture. Hence, the replication complex produces new free HCV RNA.

The newly polymerized free HCV RNA $R_{f}$ diffuses unbound as long as it does not get bound to ribosomes. A small part is sequestered directly again at the ribosomes of the local web to translate new polyprotein on-site. However, a substantial part of $R_{f}$ diffuses away at the ER surface, visible at the second (lower) sector from the right.

Once free HCV RNA reaches the subsequent ribosomal region, free HCV RNA is sequestered by the ribosomes (visible at the second upper sector from the left) to ribosomal bound HCV RNA, $R_{r}$. Hence, polyprotein *P* is translated (cf. the second upper sector from the left). Then, a new HCV RNA cycle starts also at this web region. To this end, a “wave” of HCV RNA and NSPs propagates through the cell and consumes the host factor. This property is also reflected in a previous work of ours [34].

### 3.2. Qualitative Stage and Robustness of the Model

The model developed in the present study, although largely inspired by our former study [34], resolves the details of the processes with greater refinement compared to previous. Nevertheless, it is still qualitative, rather than quantitative, as it relies on parameters that are not always derived from experimental data. This is due to the fact that—at least to the best of our knowledge—most diffusion coefficients featuring in the diffusion–reaction equations are not available in the dedicated literature. In addition, the reaction rates are usually not resolved in space, since they feature as sink or source terms in ODEs. This limitation notwithstanding, a parametric study of our model has shown its robustness for a large range of parameters, thereby leading to the conclusion that it can give plausible results also when suitable experimental data are available.

### 3.3. Experimentally Motivated Hypothesis within the Model

An interesting feature of our new model is that the mathematical form allows one to choose the diffusion coefficient of HCV RNA so that HCV RNA transport depends upon NSP concentration. This means that, within the webs, (free) HCV RNA diffusion velocity depends upon the web protein concentrations. On the ER, (free) HCV RNA transport depends on the NS5A concentration. NSPs/NS5A “shuttle” HCV RNA. The “shuttle” hypothesis means that NSP/NS5A concentration boosts HCV RNA transport for high NSP (NS5A) concentrations, while low NSP (NS5A) concentrations disable HCV RNA transport, i.e., HCV RNA is shuttled by the NSPs (NS5A). Therefore, HCV RNA may only move on such paths where NSPs are available. Our speculation is based upon experimental hints [21], which we explain in more detail in the Appendix B.2. Since this property is speculative to some extent, our model scenario incorporates also the limit case of HCV RNA diffusion which decouples from the NSP/NS5a concentrations—adequate parameter choice allows for a NSP independent HCV RNA diffusion coefficient.

Future experimental efforts may hopefully address the “boost/shuttle” hypothesis. For more detail about the current experimentally difficult question about the HCV RNA movement properties [20], we refer to Section “4.6. The HCV RNA Transport and the Web Movement” of our previous paper [34]. We further refer to some recent and relevant experimental studies that define [20,21] “The spatiotemporal dynamics of Hepatitis C Virus (HCV) RNA localisation are poorly understood” [21].

Finally, the combination of different aggregate states of the agents of the HCV RNA cycle with full spatial resolution leads to an interesting model property: The model aims to mimic the condition that NSPs only replicate their own “mother” HCV RNA. This condition is also called cis requirement condition [20,56,57]. The construction process of the RC of our model ensures that the HCV RNA may pick up web proteins exclusively close to that location where they have been created. In fact, the RC arises as a combination of HCV RNA and web protein translated by just the same HCV RNA. Indeed, the web proteins that the HCV RNA can only pick up belong to *one* (namely the same) web region: exactly that web region that is created by web proteins translated by the ribosomal region where the HCV RNA dissolutes to form RC. Hence, the web proteins of the RC have their origin within the HCV RNA of the RC. Therefore, the web proteins of the RC always translate their own “mother” HCV RNA. The “cis” requirement for HCV RNA replication is fulfilled, as assumed based on experimental data [56,57] (for more detail, see the Appendix B.1). To the best of our knowledge, this property has not yet been captured by any published mathematical virus replication model.

However, the model structure is not limited to this condition. Small model extensions would also enable replication complex formation by means of the combination of a free HCV RNA genome with NSPs, that have not been translated by this free HCV RNA genome. Such model extensions would just incorporate an additional reaction term. However, we have not performed such an extension.

### 3.4. Quantitative Evaluation in Subdomains

**Figure 6:**
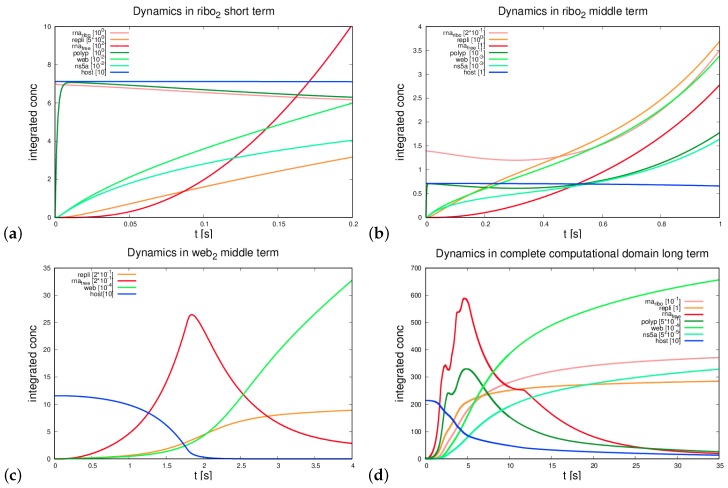
Evaluation of the integrated concentrations (Equation (13)) of the components of the basic sPDEs (Equation (5a)–(5g) with initial conditions in Equation (7a)–(7c), using the test parameters, as indicated in Table 2 and Table 3) in different subdomains: (**a**) ribosomal region # 2 ($R_{2}$) short term; (**b**) ribosomal region # 2 ($R_{2}$) middle term; (**c**) MW region # 2 ($W_{2}$) middle term; and (**d**) complete computational domain (𝒟) long term (cf. Section 2.9 and Section 3.4) Results are shown at grid refinement level 4. The graphs of this figure depict the evaluation of the integrals of the concentrations (Equation (13)) over time for different species. The time is shown at the *x*-axis. The integrals of the concentrations are shown at the *y*-axis. The integrals of the concentrations are the sum of each component within their respectively-defined region. The four graphs consider the integrals within different time scales and also within different geometric regions. The integrals are computed within the indicated regions. For example, the integral of (**a**) is computed over the ribosomal region #2, $R_{2}$, therefore the integral reads as given for this special case in Equation (12). The other regions are also indicated within the title of the graphics (**a**–**d**), cf. Equation (13). The consideration of different time scales allows for consideration of the processes at different temporal resolution, namely for considering the processes structure at special regions at the beginning of the cycle, for example how the HCV RNA translates polyprotein and how the polyprotein splits at the very beginning (cf. (**a**)). The split of the polyprotein causes the appearance of web (accumulating) protein and NS5a. In (**b**), we consider the process for a longer time scenario. In, (**c**) the consequences at the surrounding web region, i.e., the dynamics of the concentrations in the web #2, $W_{2}$, which grows on top of the ribosomal region # 2, are considered. We observe the uprise of free RNA, which is produced by the RC, until when the host factor is depleted. Then, locally, free RNA decreases again. The decrease of ribosomal bound RNA at the beginning corresponds with RC creation, while ribosomal bound RNA rises up again once free RNA is produced substantially. We note that the integral of graph (**d**) is performed over the complete computational domain D. The last graph (**d**) shows the sum of each component within the complete part of the cell we consider. We see the uprise of free HCV RNA, and we also observe the staircase similar steps when the next web region is reached. Then, free RNA is sequestered by the ribosomes, and new polyprotein arises which splits again into NSPs to continue the cycle. The host factor depletes more and more.

As described in Section 2.9, we wanted to evaluate the temporal evolution of the integrals of the concentrations over distinct subdomains. Each curve in Figure 6a–d refers to the integral of the concentration of a species present in a given subdomain (as defined by Equation (13)). For example, the curve labelled with ${\mathrm{rna}}_{\mathrm{ribo}}$ in Figure 6a shows the time evolution of the integral of the concentration $R_{r}$ of the ribosomal bound HCV RNA computed over the ribosomal region $R_{2}$ (cf. Equation (12)).

In detail, we evaluated the evolution of the integrals of the species’ concentrations within the first ribosomal region, i.e., where the initial ribosomal bound HCV RNA concentration is located (ribosomal region number 2, $R_{2}$). The results of these evaluations are reported in Figure 6a,b for the short and middle term time scales, respectively. For the middle term, we also show the evolution at web region number 2, $W_{2}$, which is attached to the ribosomal region $R_{2}$ (cf. Figure 6c). Figure 6d reports the evolution of the species’ concentrations within the complete computational domain for the long term evolution. Whereas the integrals of the concentrations of the free HCV RNA, $R_{f}$, host factor, *H*, and polyprotein, *P*, decrease in time due to the presence of sink terms featured in Equations (5b), (5f), and (5g), the integrals of $R_{r}$, *W*, *C*, and *N* increase in time. This behavior is due to the fact that the reaction terms of Equations (5a) and (5c)–(5e) are positive over the long time scale, and no degradation term has been accounted for. We emphasize that these terms have not been neglected on the basis of biological considerations, but only because we had no available experimental data for them. However, should such data become accessible, the corresponding sinks could be easily taken into account in the model and implemented in the UG framework. Our choice is motivated by the need of focussing our attention on aspects of the dynamics that, to the best of our knowledge, have not been addressed so far in the literature.

For example, with reference to Figure 6d, we notice that the integrals $I_{R_{f}}\left(D\right)$ and $I_{P}\left(D\right)$, associated with the free HCV RNA and the polyprotein, respectively, exhibit rather marked local peaks at given instances of time. The decrease between two subsequent peaks is due to the fact that both species are partially transformed. Indeed, the free HCV RNA is partially bounded to the ribosomes thereby generating $R_{r}$, and the polyprotein is cleaved into the NSPs, namely web-protein and NS5a. A similar result has been reported in our former paper [34]. However, the new model allows for the visibility of the local decrease of free RNA when free RNA reaches a new ribosomal region and binds to the ribosomes.

For the integrals over the complete domain, we show intensive refinement test studies in the Appendix F.1.

#### A Test for the Model Calculations

Looking at the evolution of the concentrations within the considered subdomains, one could conclude that the translation of the polyprotein, the formation of the replication complex, or the polymerization of new free HCV RNA are allowed to continue also in the absence of a sufficient amount of the host factor. This, however, cannot be the case, since it would contradict the model principles. Moreover, a more careful analysis of the simulations, performed for example by conducting a parametric study on the model, highlights the physical reason for which the above-mentioned processes should stop when the concentration of the host factor goes below a certain threshold.

In fact, by switching off the diffusion coefficient of the host factor on the ER surface, one forces this species to diffuse over the web regions only, thereby preventing it to enter the web regions from the surrounding ER. (To “switch off” the diffusion coefficient means to set it to zero within a special region.) In this case, we found that, once the host factor is depleted, all the processes whose evolution depends on the availability of the host factor stop immediately. This result is consistent with what we reported in our former study [34].

### 3.5. Profiles of the Distribution of the Concentrations Across One-Dimensional Trajectories on the ER Manifold

We evaluated the concentration profiles of different components along one dimensional trajectories on the ER manifold. (The trajectories we used were displayed in Section 2.10.) As described in detail within Section 2.10, we evaluated free RNA and host factor on the long ER and web crossing path, whereas we evaluated all concentrations that appear within the corresponding sub paths for the case of the small region around a special web, i.e., the path which splits into four sub parts, as explained in Section 2.10.

**Figure 7:**
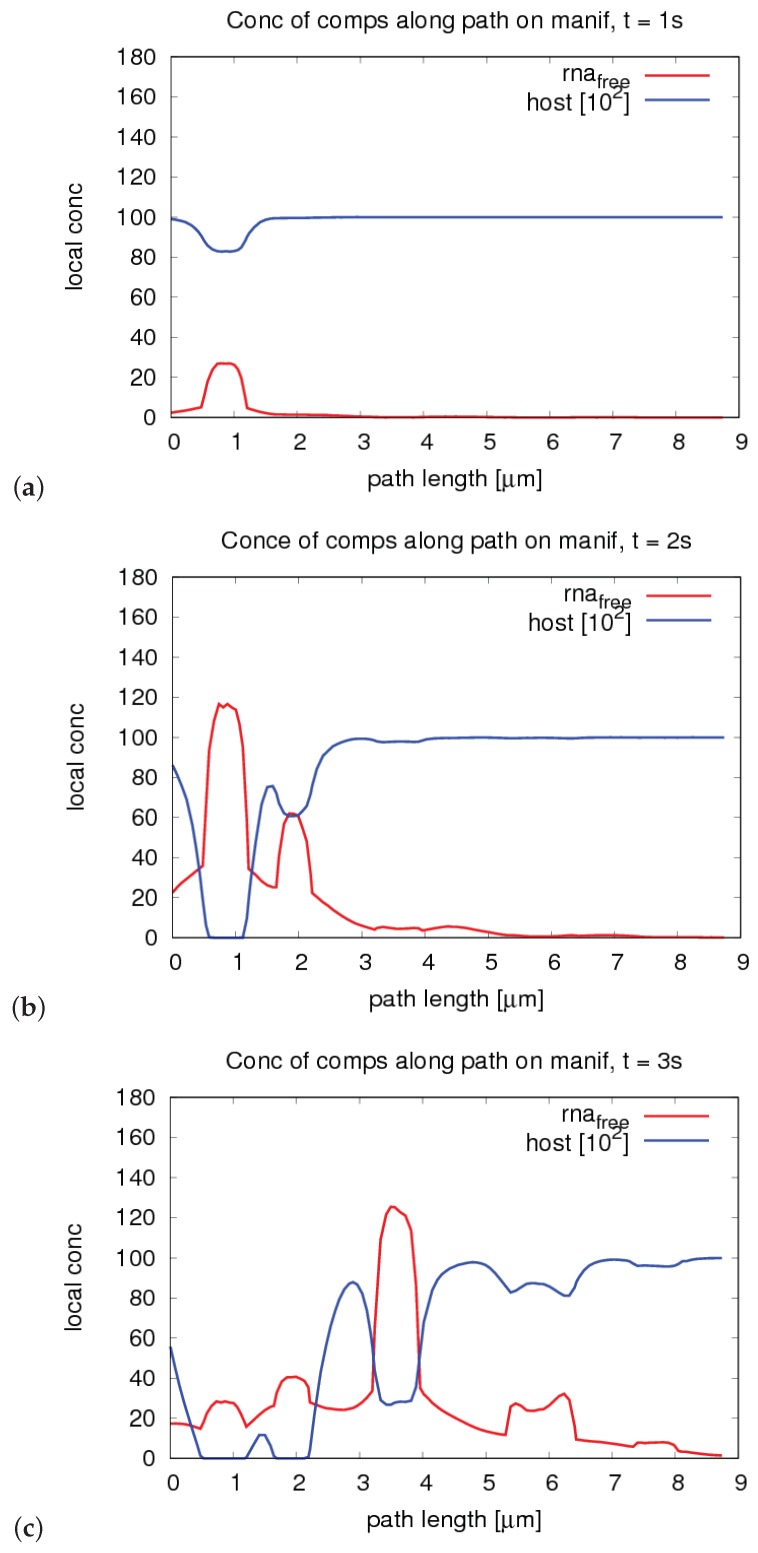

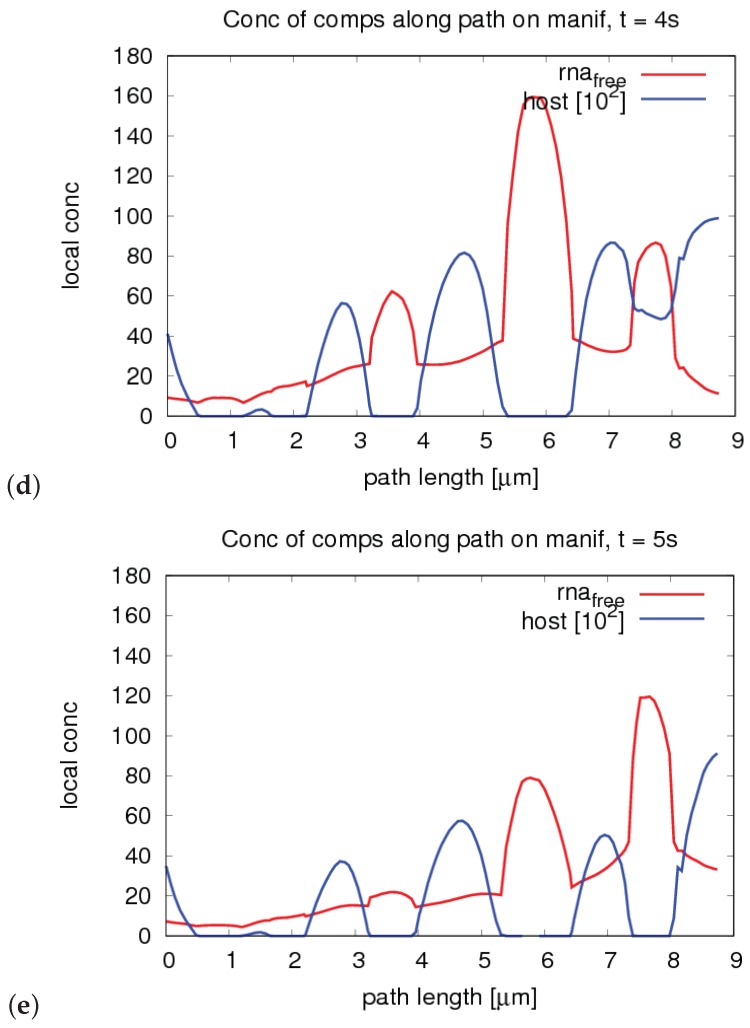
1D profiles of concentrations along the long path, cf. Figure 2. (For details, cf. Section 3.5.) One sees clearly how the “peak” of free HCV RNA moves through the cell at different time points t=1,2,3,4,5s. ((**a**): t=1s, (**b**): t=2s, (**c**): t=3s, (**d**): t=4s, (**e**): t=5s). Note: The path length seems to be similar on the ER surface between two neighboring webs and from the (spatial) beginning of one webs until its end. However, this is because we cross the webs on their respective longest path “on the top”. The direct distance from the beginning of a web to its end is smaller compared to the distance between two webs. Results of grid refinement level 4. Consider also Figure 9, where we mark the peaks and relate them to their corresponding web “number”.

**Figure 8:**
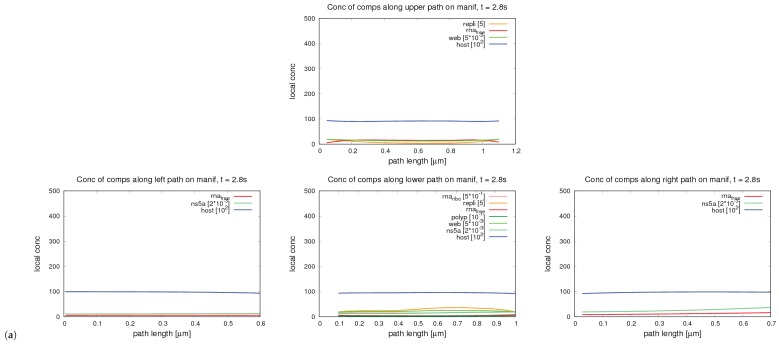

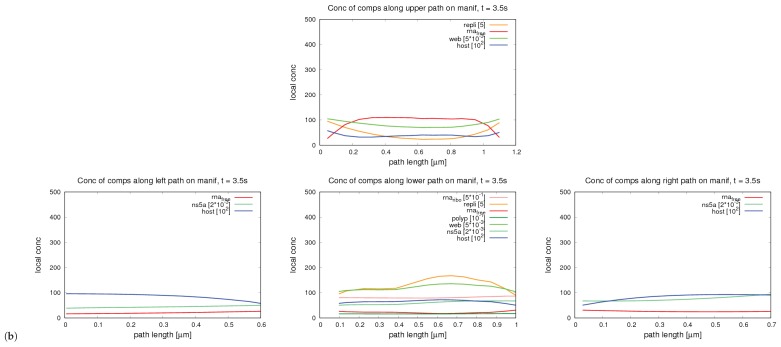

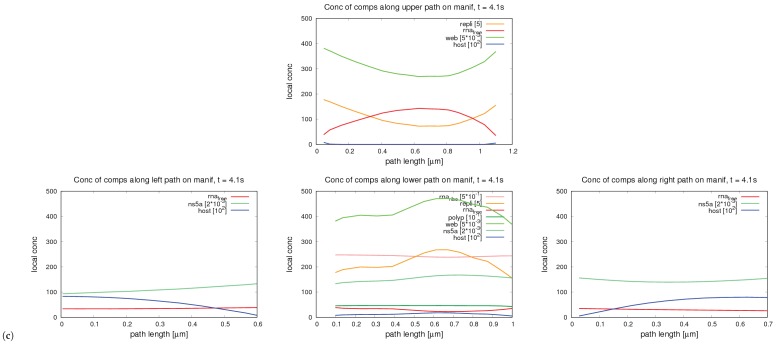
1D profiles of concentrations along the short path (as shown in Figure 3) around a single web. (For details, cf. Section 3.5.) One sees clearly how the concentrations are strongly enhanced around and especially within the web: first time point of observation (**a**): t=2.8 s; second time point of observation (**b**): t=3.5 s; and third time point of observation (**c**): t=4.1 s. Note: Ordering of the figures corresponds to the spatial order of the sub paths as displayed in Figure 4. (Each single sub graphics of each sub figure corresponds to one sub path, i.e., the time evolution of the concentrations of each sub path is reflected by means of the values shown in the corresponding sub graphics of each sub figure. The ordering of the single graphics belonging to a certain time point of evaluation corresponds to the sub paths which are depicted in Figure 4. To this end, for example, the left sub graphics of sub figure (**a**),(**b**) and (**c**) belongs to the left sub path at the time points t=2.8 s, t=3.5 s, and t=4.1 s.) Note that the concentrations belong to one single web and its close environment in this case, since the geometrical space observed refers to a single web and its close environment. The peaks of concentration are hence related to special locations inside the web under consideration. (Results of grid refinement level 4.)

We show the time evolution for both cases for time points within windows where there are important changes visible. The free HCV RNA “flow” is coming originally from the right and continues to the left. In Figure 7, we show concentration profiles along the long path, as depicted in Figure 2. An example of the four sub paths around one web, as depicted in Figure 3, is shown in Figure 8.

**Figure 9:**
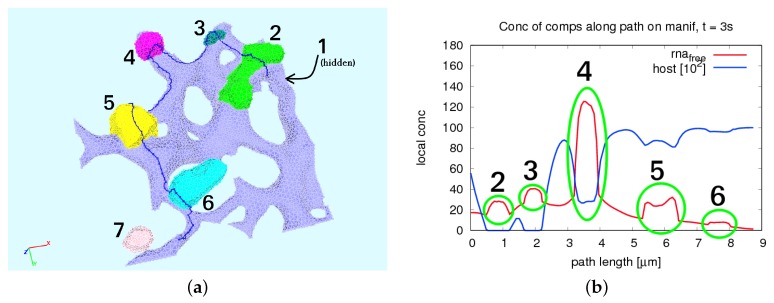
Web regions and their reflection within the evaluation of the concentrations of free HCV RNA and host factor along the 1D trajectories as introduced in Figure 2. Web region #1 is not visible from the given perspective. Therefore, we also did not include it within the path of evaluation. In the **left** picture (**a**), we show the numbering of the web regions, whereas in the **right** picture (**b**), we see the reflection of the web regions along the trajectories by means of the changes of the concentration values. We choose the time point t=3 s to demonstrate the relation of free HCV RNA peaks within the webs (and host factor depletion), since, at this time point, all free HCV RNA peaks corresponding to its respective web region are at least somewhat visible. We mark the free HCV RNA peaks with a green circle. The host factor depletion centers are visible at the same locations of the path length (i.e., *x*) axis. Compare also Figure 7 for time evolution along this trajectory.

The relationship of peaks and corresponding web regions is shown in Figure 9 for a special time point in the case of the “long” path, corresponding to the evaluation shown in Figure 7. Since the graphs of the concentrations along the “small” path (cf. Figure 8) show the evaluation around one special web (web # 5 using the notation of Figure 9, left picture), the peaks are related to the same web of course in this case. Hence, we do not show additional graphs in this case.

We denote that we compute the concentrations only at those node points that appear also at base level. This restriction is due to comparison reasons for refinement test checks. Therefore, in part, the concentration profiles make the impression that they are in part non-smooth (i.e., incorporating discontinuous derivatives at certain points). However, this is only due to the restricted point number of evaluation for the sake of comparison. The entire refinement tests are displayed in the Appendix F.2.

### 3.6. Process Analysis—Properties of Our New Model

We have developed a new population dynamics inspired sPDE model which reproduces the HCV HCV RNA replication cycle in a spatially-resolved manner.

Strikingly, our new model reflects the experimental reality in more detail than in our previous study [34]. We have enlarged our former model [34] (which we recapitulate briefly in Section 2.4) by means of two major model properties:

- We introduce population dynamics elements for the structure of diffusion and reaction coefficients. To the best of our knowledge, this feature had not yet been used within computational virology at an intracellular level. (The population dynamics coefficient structure could also contribute improvements to state-of-the-art non-spatial kinetic models of intracellular virus replication.)
- Currently, our spatial model harbors the possibility, that an agent may appear within different aggregate states, e.g., chemically bound or unbound. Similar states are already part of some ODE models (see, e.g., [8,18]).

The utilization of these new features causes the main properties of the HCV RNA replication cycle to be resolved very precisely. Some of the new features have their origin in the use of population dynamics coefficients, some in the use of different aggregate states for the agents, while some arise only because of the combination of both properties in combination with the spatial resolution. The following features arise within our new HCV RNA cycle model.

- The HCV RNA presently harbors different states for each action, namely for:
  – translation of viral proteins—ribosomal bound HCV RNA $R_{r}$;
  – polymerizing new HCV RNA—replication complex *C* formed by a combination of HCV RNA and web proteins
  – free HCV RNA $R_{f}$—may diffuse within the MW and on the ER surface.
- Different NSPs arise from the viral polyprotein: namely
  – MW (accumulating or building) web protein; and
  – freely diffusing NS5a.
- Population dynamics inspired diffusion and reaction coefficients:
  – replace multilinearity (which is valid only in the case of small, but also not too small, concentrations) by coefficients that are valid at all scales of concentrations;
  – enable the mimicking of quasi-single particle processes within the framework of continuum models, thus cause that the interpretation of concentrations gets close to the idea of expectation values;
  – enable one to model the replication complex state as a combination of a clearly defined number of web proteins together with a HCV RNA; and
  – enable one to model that the HCV RNA transport is “boosted” or disabled by NSP/NS5a concentration, depending on the NSP/NS5a concentrations, i.e., NSPs/NS5a may “shuttle” HCV RNA (but adequate parameter choice disables this speculative, even though experimentally encouraged [21,58,59,60] dependency) (cf. some literature citations that favor such a property; Appendix B.2).
- The combination of all aspects of the new model allows for the likely first mathematical model which mimics the requirement that an HCV RNA likely may only be replicated by its own “child” NSPs [56,57], i.e., by NSPs which arise from translation exactly by this HCV RNA (cf. the Appendix B.1). This property is also called “cis” requirement for HCV RNA replication.

Here, we noted in brief the properties of the new model in a more qualitative manner. In the Appendix C, we discuss in detail the representation of the new model properties within the mathematical structure of the equations.

## 4. Discussion

Our in silico approach mimics the interplay of HCV RNA, non-structural viral proteins, and a host factor. This in silico technique portrays HCV RNA reproduction in a 3D spatiotemporal resolved manner. Our sPDE equations are highly related to the realistic reconstructed ER geometry surfaces, which play a major role in plus strand HCV RNA virus replication [4].

Whilst our previously published models restricted the reactions to their appropriate geometric regions [34] and enabled unexplored insights into viral replication dynamics (e.g., related to the relation of form and function), these basic models harbored various aspects that required improvement. Therefore, the aim of this study was to extend the comparatively basic model of our former paper [34]. (The computational grid we used in this work is the same as what was elaborated on in the former study [34]). The main features of the model extensions of this study were to incorporate more realistic qualitative elements, which mimic better known experimental properties. Hence, we introduced various intermediate aggregate states for the agents of consideration. Furthermore, the parameters for the diffusion and reactions coefficients are modeled in a nonlinear form inspired by population dynamics replacing multilinear functions of the concentrations (as they are state of the art, although they are valid only for the case of small concentrations). Both new properties, especially their combination with spatial resolution, allow for a very realistic description of the dynamics.

In this section, we discuss the properties in a more qualitative manner. For a more technical discussion, we refer to the Appendix D.

### 4.1. Properties of the New Model to Address Former Restrictions

In Section 3.6, we report the advantages of the new model. Here, we analyze the reasons for the progress induced by the new model in a rather descriptive way. In contrast to this, in the Appendix D.2, we perform a highly technical analysis of the improvements.

The major backbones of the new model properties are based on the use of different aggregate states for the HCV RNA cycle agents, and the use of population dynamics.

The introduction of intermediate aggregate states allowed for quasi-realistic descriptions of the single events. In particular, the uncertainty concerning the action of the HCV RNA was addressed in this study by means of the introduction of different aggregate states for the HCV RNA. Only free HCV RNA may diffuse inside at the ER surface, only ribosomal bound HCV RNA can translate polyprotein, and only RC bound HCV RNA can be replicated. Biologically-incompatible situations resulting in HCV RNA being translated as it diffuses away and being copied at the same time are not possible in the improved model.

Transitions between the different aggregate states are described by means of clearly defined reaction terms, which, for example, may cause free HCV RNA to get “captured” by ribosomes.

In this study, we introduced elements of population dynamics for the structure of reaction coefficients. This indicates that, for the limiting case of small concentrations, processes effectively may not take place. Only once there is enough concentration available, the process is “switched on”. One can generate transitions of function shapes ranging from slow, soft, and smooth schemes up to nearly staircase-like shapes. (We demonstrate this property concerning the shape of some population dynamics type functions for test cases shown in the Appendix I. Indeed, depending on the choice of the parameters entering the population dynamics type functions, the shape of the population dynamic type functions may take forms of rather smooth functions or rather sharp functions which show quasi-step-like transitions).

The feature of population dynamics switching on and off processes depending on the concentrations of the agents is important for the construction of the RC and for the translation of polyprotein by ribosomal bound HCV RNA. The combination of a RC is always practically the same: One HCV RNA always is bound chemically to a defined amount of web proteins and forms the RC. Descriptively, one can imagine the RC construction process as if one (ribosomal bound) HCV RNA “picks up” a defined amount of web proteins *W*. This combination causes the creation of the replication complex *C* and is possible only if enough HCV RNA and web proteins are available. We assume that also, within in vivo/in vitro cell experiments, the number of NSPs that are involved within the replication of one HCV RNA is more or less always similar. Therefore, our description approximates biological reality. Furthermore, the polyprotein translation may start within our new model only if a substantial amount of HCV RNA is ribosomal bound, but not before. Finally, the host factor consumption for the construction of one new HCV RNA is now always practically the same and does no longer depend on the concentration of available web proteins.

Population dynamics, however, does not only allow for the “switching on” of a process. In fact, it further restricts the process strength (diffusion or reaction) even in the limit of infinite concentration to a clearly defined value. This ensures that reaction strength does not increase in a biologically unsound manner. Reactions may start only or continue to take place efficiently once a substantial concentration of the corresponding agent is reached, or as long as a substantial concentration of an agent is still present. This property ensures that a reaction may start or continue only if a substantial concentration of the concerning component is reached.

The diffusion coefficients of some components are modeled in population dynamics form such that the concentration of NSPs modulates the possibility to diffuse. Therefore, this form of the diffusion coefficients suggests that diffusion is only possible once sufficient NSPs are available at a spatial point. (The diffusion coefficients have a defined limit for “infinite” NSP concentration, which is practically reached for realistic concentrations.) This property ensures that some agents may only diffuse to such places where NSPs are already located. Therefore, diffusion of free HCV RNA or RC into a web region where the web protein is not dense is no longer possible. This guarantees the theoretically important property that free HCV RNA may not diffuse into other (geometrically defined) MW regions as long as there is not a substantial amount of web proteins that is already accumulated there. The lack of accumulated web proteins corresponds to a geometric MW region where the functional MW has not yet grown.

To this end, choosing model parameters of the reaction and diffusion coefficients that are suitable, our new population dynamics model inherits even the simple linear case as limit. This enables the choice of a simpler model within the given framework.

### 4.2. Spatiotemporal Resolved Evaluation of the Concentrations

As in the case of the predecessor of this model [34], we may compute the concentrations at each spatial point of the computational grid at each time. We demonstrate this property in Section 3.4. We depicted the evolution of the concentrations of the different aggregate states of the agents of our model integrated within different subdomains and over the complete computational domain. (We also demonstrated the grid convergency of our results; cf., the Appendix F.1). The latter values should serve to compare to experimental values like those published previously [42,43] once the computational domain is extended to complete hepatocytes. Experimental approaches which complement our ansatz in a more refined way can be found in more recent experimental studies (see, e.g., [20,21]).

#### 4.2.1. Simultaneous Observations In Vitro–In Silico “Loupe”

The simulations of our model reproduce the experimentally observed in vitro effects that take place when a hepatocyte cell gets infected with HCV. In particular, the simulations reproduce these effects in a detailed spatiotemporal resolved manner. Quantitative conclusions are possible due to the complex dynamic model. We briefly recapitulate the major effects that can be observed simultaneously in our in silico simulations and which, in principle, can be observed and compared with in vitro experiments: An HCV RNA genome binds to ribosomes and induces viral polyprotein translation. The polyprotein cleaves into different nonstructural viral proteins (NSPs), namely those that form the web regions and others that diffuse away on the ER surface. In experiments as well as in our simulations, one further observes that HCV RNA moves into separable web regions. HCV RNA captures NSPs of the web region forming replication complex (RC) which polymerizes new HCV RNA. Free HCV RNA diffuses away to other ribosomes to start new polyprotein translation at another region of the cell. Here, the modeling approach allows a much more detailed view on these processes as currently available experimental results, but are in agreement with present experimental studies forming the basis of the HCV RNA replication theory (see, e.g., [1,4,6,61]).

The current study forms the basis of a model for which subsequent in vitro experiments could seek to augment. Our simulations resolved the processes at such a small scale in time and space that they will provoke advanced experimentation to validate details of our model, such as: Do the growth of webs spread from single points of origin, or does this process happen randomly? Our detailed model approach is an important and complex step that may allow for further validation from future experiments that provide insight into the dynamics of proteins within cells (in vitro models or even cells originating from an infected liver). Such experiments would also allow further modifications and adjustment of the spatiotemporal model presented here and can provide a quantitative assessment of the model parameters, but are beyond the aim of the present study.

#### 4.2.2. Profiles Of Concentrations Along 1D Trajectories On The ER Surface

To this end, we note that we evaluated profiles of concentrations along selected one dimensional curves on the ER surface (Section 3.5) and we have shown grid convergency of these results (Appendix F.2). Such results could serve for comparisons with, e.g., fluorescence experiments.

### 4.3. Interpretation of Local Concentrations

Within our population dynamics coefficient structure, one can choose coefficients so that polyprotein translation may only take place if there is at least one HCV RNA bound at the ribosomes, or RC may only be constructed once there are many of web proteins available.

This necessity is better understood if we consider the amount of ribosomal bound HCV RNA concentration (or of available web proteins for RC construction) in a similar way to the expectation value of the spatial variable of a state, e.g., in quantum mechanics, i.e., if we interpret the concentration of ribosomal bound HCV RNA as some sort of probability that a comparable amount of whole-number HCV RNA is bound. For example, a “quarter” of a HCV RNA genome cannot translate polyprotein.

Indeed, one central property of HCV RNA movement and binding in de novo infections is that relatively few HCV RNA genomes are introduced into the host cell during initial infection with HCV [43]. This property indicates that a continuum model of HCV RNA can lead to uncertainties such as: How can a concentration of HCV RNA cause translation if the concentration is much smaller than a single genome (i.e., integrated over the surface of the ribosomes)?

The population dynamics form of the reaction coefficients indicates that translation may only start if a relatively clearly defined amount of HCV RNA is bound at the ribosomal surface. In the case of small concentrations of HCV RNA at a ribosomal region, it is not very likely that a HCV RNA genome is attached to the ribosomal region. Then, translation is effectively not possible.

The introduction of elements of population dynamics into reaction and diffusion coefficient structures of computational virology allows continuum model properties to link to a form of quasi-single-particle model properties. For the transition from lower to higher concentrations, population dynamics allows for quasi-transitions from quasi-few-particle descriptions within the diffusion–reaction parameters to real continuum models for high concentrations. Population dynamics allow for “slow” smooth transitions and, depending on the parameter choice, quasi sharp transitions between continuum and quasi single particle states, respectively. Population dynamics enable quasi-transitions between continuum model descriptions and elements of single particle modeling, however all these effects are described within a continuum model framework. Our ansatz represents a method that emulates few-particle-system properties for small concentrations and entire continuum model properties for high concentrations.

### 4.4. Summary—Progress Induced by the New Model

The combination of population dynamics and different aggregate states with fully 3D spatial resolution upon realistic reconstructed geometries allows resolution of the major principal aspects of the spatial dynamics, and is more consistent with the biology of the HCV infection cycle.

For example, the “picking up” of a defined number of web proteins by one HCV RNA to form the RC is closer to experimental reality than the previous state-of-the-art models, i.e., the multilinear reaction models.

The combination of population dynamics and spatial resolution further allows for the modeling of the speculative hypothesizes like HCV RNA shuttling by NS5a and the “cis” requirement for HCV RNA replication [20,56,57]. Surprisingly, the cis requirement appears in a rather natural way upon the adaption of the model to experimental reality concerning basic aspects—a 3D realistic geometry combined with different aggregate states. We did not impose the condition directly. Indeed, we did not even try to impose it. It arose once the model got sufficiently realistic. We could delete this condition again by adding additional reaction terms, but we omit such a step at this stage. Indeed, the property that arises from our model is a type of quasi description of this condition. Interestingly, this property arises within the framework of a continuum model. Such a realistic mathematical description is only possible within spatial resolved models.

This model is an approach to describe different experimentally observed facts and experimental-based hypothesis. Such effects (e.g., NSP concentration dependent HCV RNA transport, constant relation of HCV RNA and NSPs within RCs, and HCV RNA replication cis requirement) were not captured within ODE models, to the best of our knowledge.

### 4.5. Remaining Restrictions of the New Model and Future Model Extensions

There remain some restrictions concerning qualitative aspects of the model.

Particularly, the spatial dynamics of the host factor(s). (In reality, likely far more than one host factors are involved in the process.) We assume that the host factor movement is not restricted in surface movement as it is modeled at the moment. In addition, the spatial dynamics patterns of the HCV RNA movement remains unsolved and respectively challenging also within the experimental context [20,21]. Thus, some questions are challenging for the mathematical side as well as for the experimental side (cf. Section “4.6. The HCV RNA Transport and the Web Movement” of our former paper [34]).

The simplification—that the MWs are resolved only at the surface and not within the volume—is less relevant because of scale arguments, since volume and surface are comparable at this stage. Therefore, the overall dynamics is influenced only minor by this aspect.

The mixture of volume and surface effects is beyond the scope of this study. Future extensions should incorporate the merging of surface and volume effects (cf. our simple volume grid model [34]) concerning the transport mechanisms and properties of the different agents. This indicates to extend the “volume” PDE (vPDE) model (as introduced by us before [34]) by means of incorporating the properties of the complex surface PDE model introduced in this study. We consider this topic in more detail in the Appendix E.1.

An incorporation of degradation effects should be tested, because our present model suffers from permanent bound HCV RNA accumulation caused by the binding of free HCV RNA to the ribosomes. We did not consider this aspect in detail, because we focused upon the introduction of a new model structure. Degradation reactions are easy to incorporate within our technical framework and are already well-known within the literature.

Necessary future improvements are to use realistic values for the parameters of the model, and we are already working on this aspect. Recently, we estimated the diffusion constant of NS5a at the surface of the ER based on experimental FRAP time series data [35,36,37]. The value could be incorporated into our model; however, this only makes sense once there is also a perspective to get experimental data for at least some of the other parameters. To this end, we are looking forward to the present and near-future experimental techniques that may enable access to suitable data for such parameter fits. Such a scenario would likely allow for a rough estimation of the remaining values (i.e., maybe not a strict parameter estimation, but at least some sort of assumption).

To this end, an important topic will be the extension of the simulations to complete cell geometries rather than a subsection of a cell so that whole-cell experimental kinetic data can be compared [42,43]. The present model focuses on perinuclear zones where the cell compartments show a relatively static internal architecture [28,62]. In the long term, the extension of the geometric region of the model evaluation to the peripheral regions will likely need to take into account the random walk properties of, e.g., peripheral NS5A foci [35], as well, which are often highly motile and capable of rapid long-range traffic, i.e., to “jump around” [21,28,32,33].

### 4.6. Incorporation of Direct and Indirect Antiviral Agents

An inclusion of the mechanisms of action of antiviral components, such as interferon or DAAs in the in silico model is possible. For example, the assumed mechanisms of action of “interferon” can be incorporated into the equation set of Equations (5a)–(5g) using a reduction factor of the reaction rate causing polyprotein translation. Furthermore, a simple multiplication factor in polyprotein translation rate depending on the natural intracellular inhibition pathways induced, e.g., by interferon could account for the interferon effect. A protease inhibitor would strongly reduce the cleavage of polyprotein into the NSPs. This mechanism would be reflected in the model, in the simplest case, by introducing a multiplication factor in the reaction coefficient that describes the polyprotein cleavage. If the factor is sufficiently small, it would describe protease inhibition, which would cause the polyprotein amount to increase strongly, while the NSP amount (in our model: web protein and NS5a) would remain at a low level, causing a very slow web growth and thus preventing quick RC construction. In addition, HCV RNA transport would be more slow than in the case without drug treatment. Since we assumed a coupling of HCV RNA diffusion “speed” to NS5A concentration, the “shuttle” effect cannot take place efficiently. As a final example, a polymerase inhibitor would be modeled by means of a factor which reduces the HCV RNA polymerase performed by the RC. Hence, our model forms a detailed base of the inclusion of direct and indirect antiviral agent effects. (For the case of ODE models, there exist several studies that investigate the reaction coefficient structure in cases when the effects of antivirals are incorporated; see, e.g., [13,63,64]).

## 5. Conclusions

The current study presents an advanced 3D spatially resolved model of the HCV RNA replication cycle as restricted to a subsection of a hepatocyte represented by its major compartments, namely the realistic reconstructed ER surface together with the reconstructed membranous web regions. In particular, our new model introduces population dynamics methods into computational virology at an intracellular level and different aggregate states for the components of spatially resolved intracellular virus models. The combination of population dynamics, different aggregate states of the agents of the HCV RNA replication cycle, combined with a fully spatially resolved realistic geometric environment allows for mathematical descriptions of various biological facts and experimentally motivated hypothesis (which thus far have not been modeled). Examples of these mathematical descriptions are the clearly defined relation of the ratio HCV RNA-to-NSP number within the replication complex, or the mathematical capturing of the HCV RNA cis replication requirement and the NS5A boosted HCV RNA transport.

To conclude, our *population dynamics inspired sPDE model* of the HCV RNA cycle increases the potential for new understanding of HCV RNA replication and defines a new basis for computational modeling of virus infections, which could underpin novel future experimental approaches.

## Figures and Tables

**Table 1:** Geometry info of the 3D surface ER representation that is used for the evaluation of the population dynamics inspired sPDE model. DoF number at base level and from one to four global refinement levels in space. (The triangular elements are named “faces”.)

Refinements	0	1	2	3	4
vertices	5645	22,769	91,418	366,320	1,466,540
edges	17,124	68,649	274,902	1,100,220	4,402,104
faces	11,467	45,868	183,472	733,888	2,935,552
DoFs	39,515	159,383	639,926	2,564,240	10,265,780

**Table 2:** Basic parameter set for the sPDE model evaluations, Equations (5a)–(5g), (6a)–(6c) and (7a)–(7c). Parameters determining the basic diffusion–reaction parameters and initial conditions. We emphasize that our code is not restricted to these parameters in any way and the stability does not depend on the use of the parameters reported in this table.

	Value	Unit
$D_{R_{r}}$	0.2	$\frac{{\left(\mu m\right)}^{2}}{s}$
$D_{H}^{\left(0\right)}$	0.1	$\frac{{\left(\mu m\right)}^{2}}{s}$
$D_{P}$	0.2	$\frac{{\left(\mu m\right)}^{2}}{s}$
$D_{N}$	1	$\frac{{\left(\mu m\right)}^{2}}{s}$
$D_{W}$	0.5	$\frac{{\left(\mu m\right)}^{2}}{s}$
$D_{C}^{\left(0\right)}$	0.1	$\frac{{\left(\mu m\right)}^{2}}{s}$
$D_{R_{f}}^{\left(0\right)}$	0.1	$\frac{{\left(\mu m\right)}^{2}}{s}$
$r_{1}$	20	$s^{-1}$
$r_{2}$	10	$s^{-1}$
$r_{3}$	0.25	$s^{-1}$
$r_{4}$	1000	$s^{-1}$
$r_{5}$	970	$s^{-1}$
$r_{6}$	50	$s^{-1}$
*v*	50	
$R_{0}$	10	$\frac{1}{{\left(\mu m\right)}^{2}}$
$H_{0}$	1	$\frac{1}{{\left(\mu m\right)}^{2}}$

**Table 3:** Basic parameter set for the sPDE model evaluations. Parameters determining the population dynamics form parameters of Equations (5a)–(5g) and (6a)–(6c). We mention that the choice of i,n,p is free in this case since we have chosen $F_{0},F_{1},F_{5}=0$ (code not restricted to these parameters).

	Value
$F_{0}$	0
$F_{1}$	0
$F_{2}$	100
$F_{3}$	10
$F_{4}$	0.001
$F_{5}$	0
$F_{6}$	0.0001
$F_{7}$	0.1
$F_{8}$	1
$F_{9}$	0.1
$F_{10}$	0.1
$F_{11}$	0.1
𝔙	50
𝔉	10
𝔊	1
*i*	N/A
*j*	1
*k*	1
*m*	1
*n*	N/A
*p*	N/A
*q*	1

**Figure A1:**
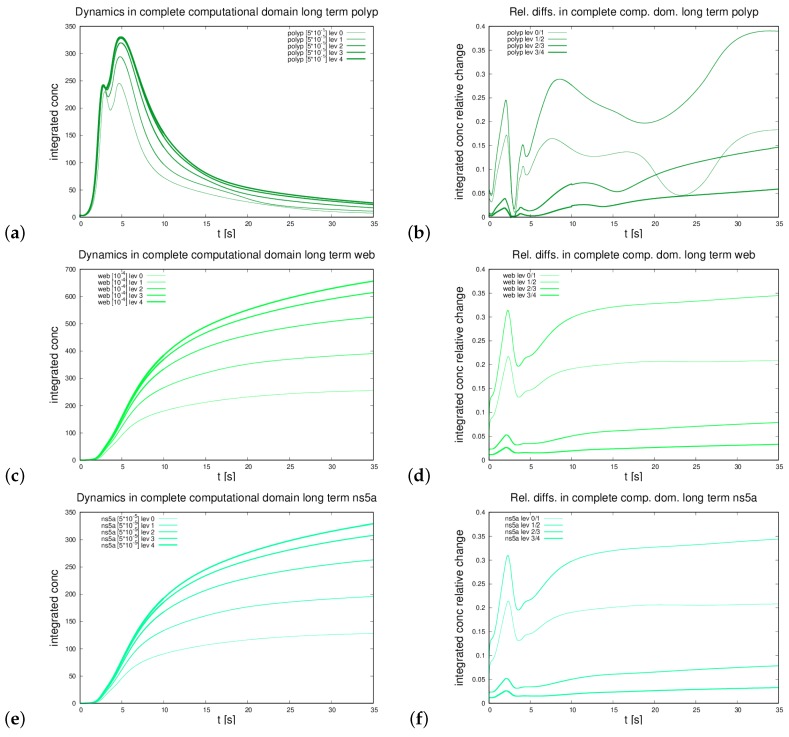
Development of integrated concentrations within complete computational domain under spatial refinement from level 0 (base level, direct reconstructed geometry) up to level 4 and relative differences respectively for concentrations of viral proteins. From top to down: Polyprotein, web protein, and NS5a. Left shows absolute values, while right shows relative differences. In detail: (**a**) Polyprotein absolute values for different grid refinement levevls, (**b**) Polyprotein relative differences for different grid refinement levels, (**c**) web protein absolute values, (**d**) web protein relative differences, (**e**) NS5a absolute values, (**f**) NS5a relative differences.

**Figure A2:**
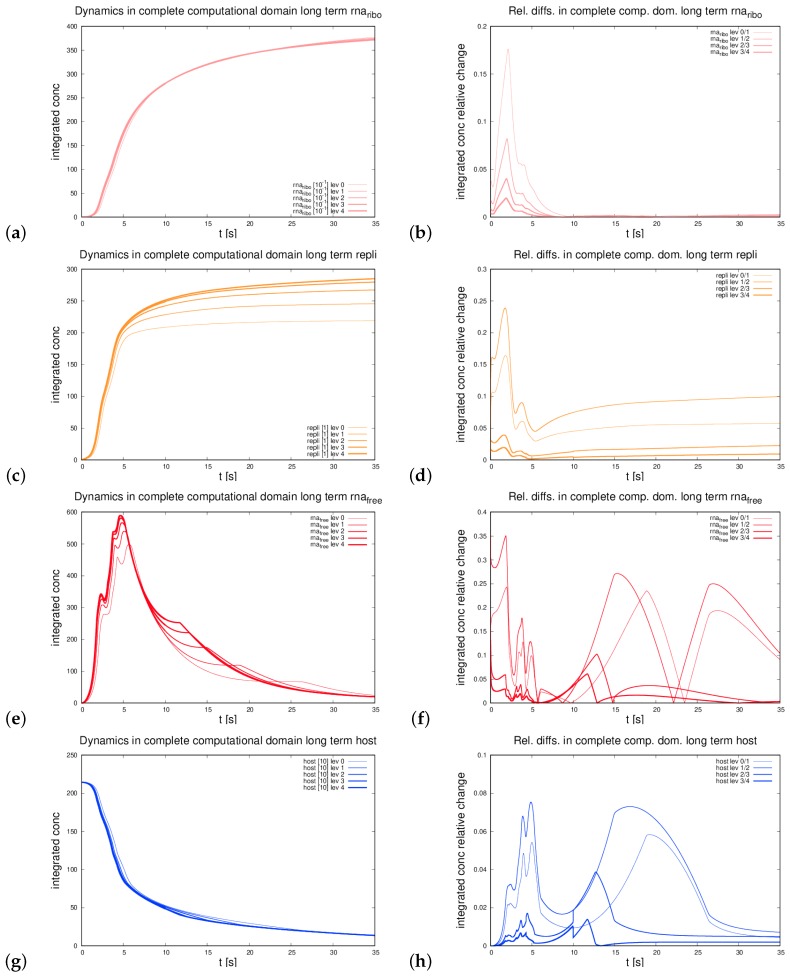
Development of integrated concentrations within complete computational domain under spatial refinement from level 0 (base level, direct reconstructed geometry) up to level 4 and relative differences, respectively, for concentrations of HCV RNA containing species and host factor. From top to down: Ribosomal bound HCV RNA, replication complex, free HCV RNA, and host factor. Left shows absolute values, while right shows relative differences. In detail: (**a**) Ribosomal bound RNA, absolute values for different grid levels, (**b**) ribosomal bound RNA relative differences for different grid levels, (**c**) replication complex, absolute values, (**d**) replication complex, relative differences, (**e**) free RNA, absolute values, (**f**) free RNA, relative differences, (**g**) host factor absolute values, (**h**) host factor relative differences.

**Figure A3:**
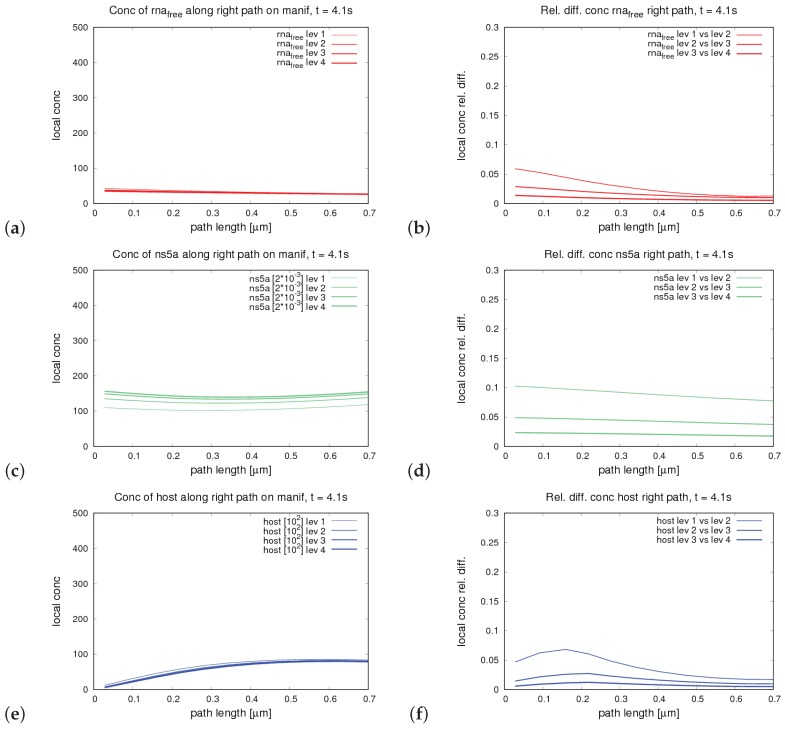
Variation of spatial refinement level for the 1D profiles of the concentrations around a web region. Right sub path. (**a**) Free RNA absolute values for different grid levels, (**b**) Free RNA relative differences for different grid levels, (**c**) NS5a absolute values, (**d**) NS5a relative differences, (**e**) host factor absolute values, (**f**) host factor relative differences.

**Figure A4:**
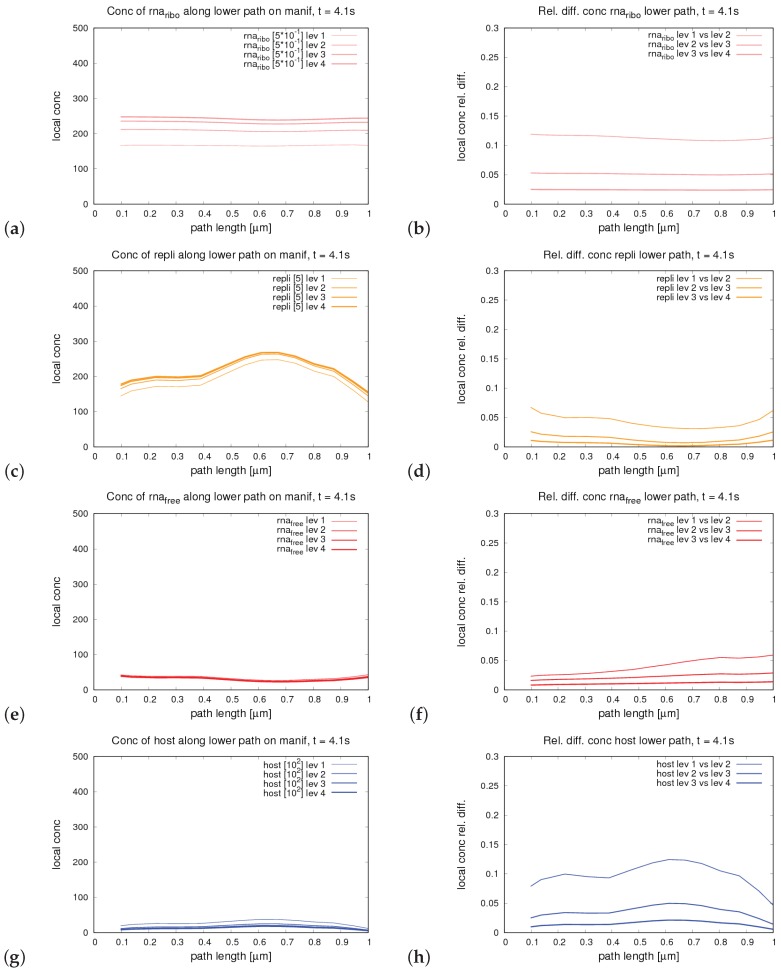
Variation of spatial refinement level for the 1D profiles of the concentrations around a web region. Lower sub path part one. Concentrations of HCV RNA containing components and of host factor. (**a**) Ribsomal bound RNA absolute values for different grid refinement levels, (**b**) ribsomal bound RNA relative differences for different grid levels, (**c**) Replication complex absolute values, (**d**) replication complex relative differences, (**e**) free RNA absolute values, (**f**) free RNA relative differences, (**g**) host factor absolute values, (**h**) host factor relative differences.

**Figure A5:**
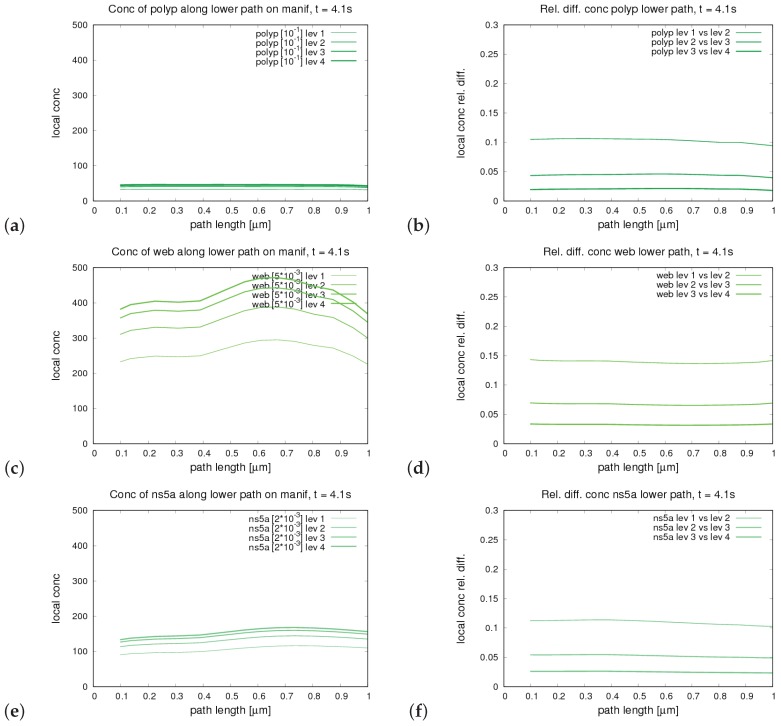
Variation of spatial refinement level for the 1D profiles of the concentrations around a web region. Lower sub path part two. Concentrations of viral protein components. (**a**) Polyprotein absolute values, (**b**) polyprotein relative differences, (**c**) web protein absolute values, (**d**) web protein relative differences, (**e**) NS5a absolute values, (**f**) NS5a relative differences.

**Figure A6:**
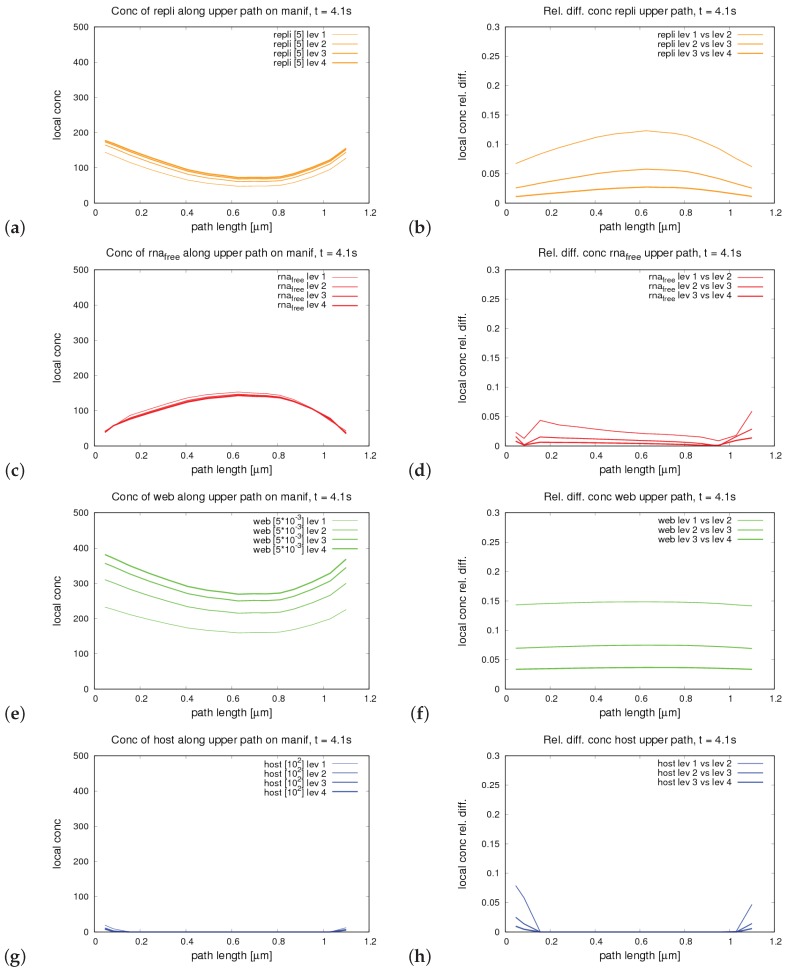
Variation of spatial refinement level for the 1D profiles of the concentrations around a web region. Upper sub path. (**a**) Replication complex absolute values, (**b**) replication complex relative differences, (**c**) Free RNA absolute values, (**d**) free RNA relative differences, (**e**) web protein absolute values, (**f**) web protein relative differences, (**g**) host factor absolute values, (**h**) host factor relative differences. (Note that, where the host factor is effectively depleted, relative differences are replaced by absolute ones.)

**Figure A7:**
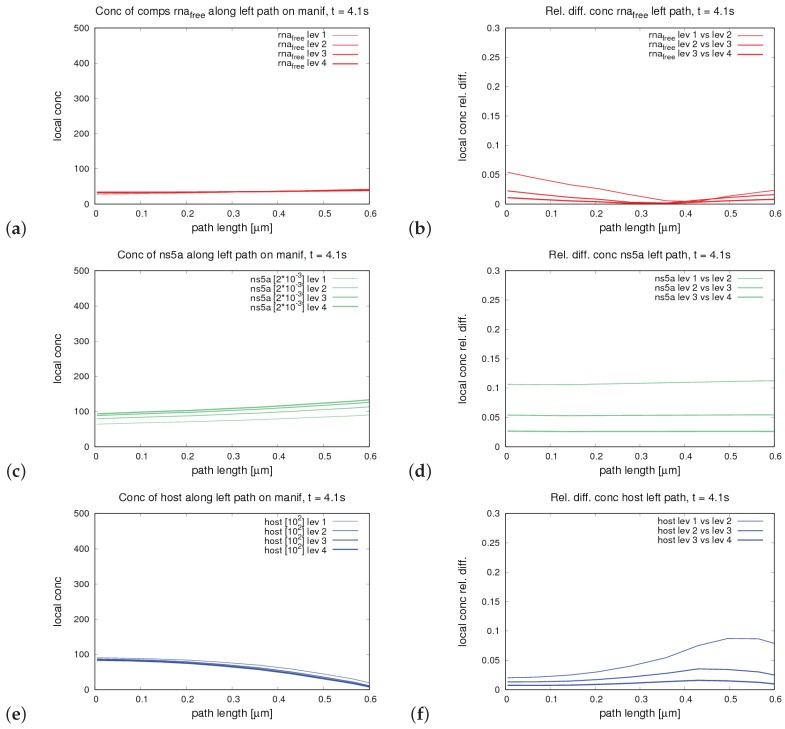
Variation of spatial refinement level for the 1D profiles of the concentrations around a web region. Left sub path. (**a**) Free RNA absolute values, (**b**) free RNA relative differences, (**c**) NS5a absolute values, (**d**) NS5a relative differences, (**e**) host factor absolute values, (**f**) host factor relative differences.

**Figure A8:**
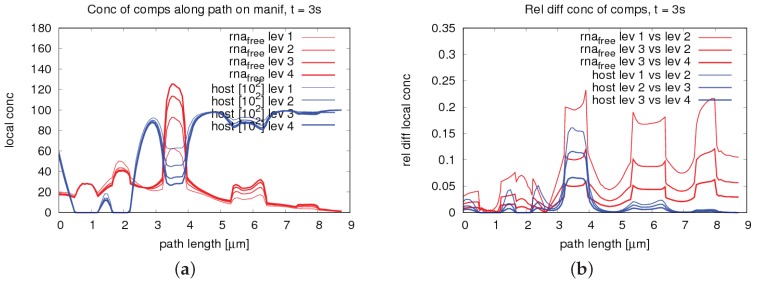
Grid convergency behavior of the concentrations of free HCV RNA and host factor along the long path around the ER and web surface at time t=3 s: (**a**) concentrations at different levels; and (**b**) relative differences.

**Figure A9:**
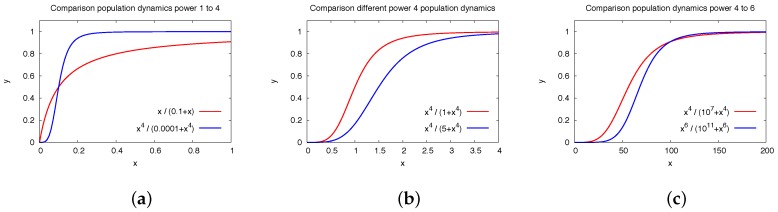
Population dynamics coefficient structure for the choice of different parameters. Plotting $y=f\left(x\right)=\frac{x^{a}}{x^{a}+b}$ for different choices of a,b. Model coefficient comparisons of possible factors: Comparison of functions with factor a = 1, 4 and 6 (cf. Appendix I). Obviously, power 4 enables sharper transitions then power 1, but power 6 does not cause further big changes. (**a**) Comparison of a=1 and a=4, (**b**) comparison for case a=4 using different values of *b*, (**c**) comparison case a=4 and a=6.

## Supplementary Materials

The following are available online at http://www.mdpi.com/1660-4601/16/3/513/s1. Supplemental Movie S1 Video: 3D surface PDE model Population dynamics inspired PDE model static view, (cf. also the Appendix A, for brief description of the simulation movie. Extended descriptions of the simulation movie can be found in the main part of this paper, cf. Section 3.1.).

## Abbreviations

The following abbreviations are used in this manuscript:

mRNA	messanger RNA
HCV	Hepatitis C virus
vRNA	viral RNA
NSP	non structural viral protein
NS5a	HCV non structural viral protein number 5
NS5aI	NS5a inhibitor
SP	structural protein
MW	Membranous web
DMV	Double membrane vesicle
RC	replication complex
DAA	Direct antiviral agent
ODE	Ordinary Differential Equation
PDE	Partial Differential Equation
sPDE	surface Partial Differential Equation
vPDE	(“volume”) Partial Differential Equation
FE	Finite Elements
FV	Finite Volumes
MG	Multi Grid
GMG	Geometric Multi Grid
UG4	Unstructured Grids version 4 [40,41]
NeuRA2	Neuron reconstruction algorithm, version 2.3 [36,47]
DoF	Degree of Freedom

## Appendix A. S1 Video: Population Dynamics Inspired sPDE Model Static View

Brief description of “S1 Video” Supplemental Movie: The evaluation of the sPDE model which is inspired by population dynamics: HCV RNA bound, HCV RNA free, polyprotein, web protein, NS5a, replication complex and host factor. View static, concentrations dynamics on ER surface are shown from different but fixed perspectives. ER in part cut and cut view from behind. Extended descriptions of the simulation movie can be found in the main part of this paper (cf. Section 3.1).

## Appendix B. Experimental Literature as Basis of Some Hypothesis

### Appendix B.1. The HCV RNA Replication and the Related NSPs

There are experimental hints to speculate if a HCV RNA can only be replicated by its own “child” NSPs, i.e., by NSPs which originate from the polyprotein translated exactly by the same HCV RNA. A book chapter [56] stated: “Due to the low and limiting number of negative-strand RNA, it is furthermore tempting to speculate that negative-strand synthesis can be initiated only once from a positive strand by a cis-acting protein complex translated on the same RNA.” Furthermore, “Since most nonstructural proteins cannot be complemented in trans, it can be assumed that RNA synthesis is initiated from a protein complex translated from the same RNA molecule.” We assume that phrase “cannot be complemented in trans” implies that the complementation has to be therefore in cis, indicating the discussed assumption. We further cite a more recent paper [20]: “The viral polymerase NS5B can only initiate RNA synthesis from the same RNA that it has been translated from (a cis requirement) [57].”

### Appendix B.2. The NSP/NS5a-Boosted Transport of HCV RNA

Our speculation is based upon experimental assumptions; we cite from an experimental paper [21]: “it was considered likely that HCV RNA may shuttle jointly with NS proteins such as NS5A” and “The ability of NS5A to bind HCV RNA is widely acknowledged as NS5A has a predicted RNA binding motif [58] and furthermore can bind HCV RNA in vitro [59,60]. It is possible that the double positive NS5A/RNA motile foci are either motile RCs and/or are involved in delivery of HCV RNA.”, and “Using such HCV genomes we have shown that both static and highly motile NS5A positive structures are enriched with HCV RNA suggesting that the motile NS5A structures are motile RCs or possible delivery vehicles for HCV RNA”.

## Appendix C. New Model Properties: Detailed Formulae and Simulation Results Analysis

We discuss step by step the consequences of the introduction of population dynamics and different aggregate states of the agents to the process structure arising from the new model:

### Appendix C.1. Ribosomal Bound HCV RNA Translating Polyprotein

The model inherits the HCV RNA replication cycle starting from the first HCV RNA attachment to a ribosomal region. The NSP translating HCV RNA is bound to the ribosomes and may not move away. (This is the opposite of our first approach [34], where we had only one HCV RNA species that was diffusing, translating and reproducing at the same time.) Viral polyproteins get translated. At other webs, polyprotein translation may only start once enough HCV RNA is “caught by” and bound to the ribosomes, realized in our model by means of the factor $A_{C}^{R}$ from Equation (10) in the reaction term of Equation (5b). (Within multilinear models, polyprotein translation is linear to ribosomal bound HCV RNA, which causes translation also for few amount of HCV RNA, even if the integrated HCV RNA concentration is far below one HCV RNA, if enough web protein is present.)

### Appendix C.2. Cleavage of Polyprotein into Various NSPs

The polyproteins cleave into various vial proteins, Equation (5b). Our model now considers different NSPs, namely a NSP accumulating NSP (called by us “web protein”), Equation (5d), and a free NSP (called by us “NS5A”), Equation (5c). The NSPs anchor to the ER surface. The web protein accumulates at the geometric MW regions. NS5A diffuses away on the ER surface.

### Appendix C.3. One HCV RNA “Picks Up” a Defined Amount of Web Proteins to Form One RC

Equation (5e) describes the replication complex *C* dynamics, namely the creation. The factor $G_{C}$ of Equation (8) inside Equation (5e) describes the preconditions for RC formation. $G_{C}$ is a product of three terms:

- $A_{C}^{R}$ from Equation (10) controls that a reliable amount of ribosomal bound HCV RNA $R_{r}$ is available.
- $A_{C}^{W}$ from Equation (9) controls that the MW has to be dense—a lot of web proteins have to be accumulated. This prevents that the replication complex may appear too early, as may happen in the case in multilinear models.
- $A_{C}^{H}$ from Equation (11) requests that host factor is available, i.e., we speculate the host factor may catalyze RC creation. (However, setting k=0, this catalyzing property vanishes again and RC construction becomes independent of the presence of the host factor.)

Once these preconditions are fulfilled, the construction of RCs (Equation (5e)) is realized as follows: Ribosomal bound HCV RNA dissolutes from the ribosomes (Equation (5a)). The amount of dissolute HCV RNA is proportional to the number of available ribosomal bound HCV RNA and to the afore-explained “precondition” coefficient $G_{C}$. The ribosomal bound HCV RNA concentration decreases in the same way as the RC concentration increases; the number of created RCs is the same as the number of that part of the before mentioned ribosomal bound HCV RNA that disassociated. Hence, the HCV RNA content of the RCs arises from ribosomal bound HCV RNA, which becomes unlocked, Equation (5a), but directly binds together with web proteins. In detail, a dissolute HCV RNA “picks up” a *v*-multiple amount of web proteins. The decrease of web proteins (Equation (5d)) involved in the increase of the RCs (Equation (5e)) is directly proportional to $G_{C}$, namely it reads $\left(-vG_{C}\right)$ with v≫1 (we have chosen v=50 for our test simulations (cf. Table 2), but it could be much higher). The population dynamics form of this process, i.e., the term $G_{C}$ from Equation (8), causes a decrease (i.e., ribosomal HCV RNA-induced catching) of web proteins effectively proportional only to the number of created RCs. This proportionality in Equations (5d) and (5e) causes that each time nearly the same amount of web proteins forms a RC together with a HCV RNA. This ensures that each RC inherits *one* HCV RNA and a *v*-fold number of web proteins (with v≫1) which care for the replication of the HCV RNA. The relation of web protein number and HCV RNA inside a RC is practically fixed. The number of web proteins inside a single RC is not proportional to the complete number of web proteins available within the cell.

We remark that the number of RCs per MW region is not limited to one within our continuum model. The total number of RCs of a MW region is calculated as integral of the concentration of *C* over the MW subdomain.

Finally, we emphasize that we do not resolve the RC in more detail, since we assume that a further resolution (e.g., introducing dsRNA, negative stranded HCV RNA or other intermediate products) has to be resolved better at smaller spatial scale. The introduction of more intermediate aggregate states at the intracellular scale bears the danger of introducing too many free parameters, thereby making it difficult to fit the model reliably to experimental data.

### Appendix C.4. Diffusion of the Replication Complex

The population dynamics inspired form of the RC diffusion coefficient, cf. Equation (6a), takes care of the fact that the RC may only diffuse to those parts of the MW regions that are already dense, i.e., where a lot of web proteins are already accumulated. This form avoids ambiguity that could arise if the replication complex is already created, but only few web proteins are bound inside the MW regions. In the latter case, the RC would diffuse into a thus far non-functional web, which would not be biologically/biophysically sound.

### Appendix C.5. HCV RNA Replication/Polymerization

The replication complex moves into the web region and polymerizes new HCV RNA there (Equation (5f)). Hence, the HCV RNA reproduction is modeled upon the aggregate state of the RC, which is described as the combination of HCV RNA with web protein in a defined and effectively fixed relation of amount HCV RNA to web protein. Hence, in our new model, a defined amount of web proteins cares for the replication of one HCV RNA as it is likely the case in experiment. The introduction of such a clearly defined aggregate state is only possible using population dynamics. Therefore, the use of population dynamics could be indicated also for ODE models of HCV replication.

### Appendix C.6. Replicated HCV RNA Diffuses Freely at First

The new HCV RNA diffuses outside the MW regions (Equation (5f)). One part binds again to the ribosomes to translate new polyprotein on-site, while another part diffuses away at the ER surface to search for other ribosomes. Once this free HCV RNA finds other ribosomes, it attaches and starts the translation of polyprotein causing a new HCV RNA replication cycle at another (geometric) MW region.

### Appendix C.7. Diffusion (Coefficient) of Free HCV RNA—-NSPs/NS5A Concentration “Boosts”/Disables HCV RNA Transport

In our model, the diffusion coefficient of the free HCV RNA depends upon the concentration of web protein in the MWs and on the concentration of NS5A on the ER surface (cf. Equation (6b)).

The population dynamics inspired form of the diffusion coefficient of the free HCV RNA, Equation (6b), causes that the free HCV RNA may only diffuse to those places where enough NSPs are available, i.e., either where there is enough web protein accumulated (i.e., within the MW regions), or where there is enough NS5A (i.e., on the ER surface). We speculate whether the transport of HCV RNA is driven by the NSPs (web protein inside the MW regions and NS5A on the ER surface). Since this idea is still to some extent speculative, adequate parameter choice disables this model property, i.e., a NSP independent HCV RNA diffusion coefficient is part of our model scenario as well. Indeed, we may switch off the “boost” property easily again by setting $F_{8}=F_{9}=0$. Such a choice would cause that the diffusion coefficient of the free HCV RNA decouples from the concentrations of web protein and NS5a. In this case, the HCV RNA diffusion decouples again from the NSP concentration, i.e., also this simpler case is incorporated within our model framework. However, by switching on the corresponding constants, NS5A concentration boosts HCV RNA transport and disables it for low NS5A concentrations.

### Appendix C.8. HCV RNA Polymerization—Host Factor Consumption

Within our new model, also the consumption of host factor per produced HCV RNA is more or less the same independent of the available concentration of web protein, host and HCV RNA (cf. Equation (5g)). Otherwise, the model would not be biophysically sound, thus valid only for small concentrations of all contributing agents. Multilinearity is the current state of art within ODE models also at this point. We also used this first simple approach within our former model [34].

### Appendix C.9. Aiming the Condition that NSPs only Replicate Their Own “Mother” HCV RNA—cis Requirement

As discussed before in detail, the RC is always constructed by a special combination.

A HCV RNA only picks up web proteins from that ribosomal region where it has translated them. The thus formed RC is always a construct of HCV RNA and web proteins that originate from *the same* HCV RNA that picks them up. Hence, the HCV RNA is always polymerized by a RC that is constructed by HCV RNA and NSPs that were translated before by that HCV RNA, which now is captured within this RC. This means that the RC always reproduces that HCV RNA from which it originates.

Therefore, another very interesting property of the model we propose in this study is that the combination of the introduction of spatial resolution and different aggregate states of the agents allows for a mimicking of the condition that NSPs may only reproduce their “mother” HCV RNA.

## Appendix D. Technical Discussion of the New Model Properties

Let us confront the restrictions of our former models [34] and the advantages of our new model in detail:

### Appendix D.1. Restrictions of our Former Models

The central restrictions refer to the topics “multilinear coefficients” and to the concept “one component–one concentration”. For example, multilinearity of the HCV RNA production causes that the HCV RNA replication consumed much more host factor, as more HCV RNA, host, and web protein were available. This seems to be biophysically not sound and can be valid only for the case of small concentrations. Further, we distinguished the role of each component (HCV RNA and NSPs) only by means of its spatial location. Therefore, our previous model was unable to capture the biological evidence according to which a substance that is, e.g., translated, cannot be simultaneously reproduced. However, “a single viral (+) strand RNA can only be involved in either translation, replication or packaging at a given time, and the switch from one process to another has to be regulated” (citation from an experimental paper [20], cf. [38]).

We consider the two major restrictions in detail.

#### Appendix D.1.1. Multilinear Reaction Coefficients

The multilinearity we used for the reaction terms [34] (recapitulated in Equations (2a)–(2d)) is based directly upon the ODE models of the state-of-the-art type [8,18]. However, multilinearity is valid only for the case of small concentrations. This argument holds true, for example, for the reproduction of the HCV RNA. Thus far, HCV RNA polymerization was modeled using multilinear reaction terms, i.e., HCV RNA polymerization depended multilinearly upon various other concentrations. Further, the HCV RNA replication consumed much more host factor, as more HCV RNA, host, and web protein were available. However, one may assume that the polymerization of *a single* HCV RNA always asks for about a similar amount of energy and host factor (cf. Equation (5f)).

Of importance is further that the HCV RNA polymerization does not start if there is only few HCV RNA concentration available (i.e., a concentration that is so small that it corresponds to the absence of any HCV RNA genome) and breaks once the host factor is practically depleted. In addition, these features are violated to some extent in multilinear models. In the former multilinear models of type RWH (Equations (2a)–(2d)), HCV RNA reproduction only breaks down once the host factor is completely exhausted. The simple multilinear proportionality causes also that a high web protein concentration increases HCV RNA replication and replication complex constructions [8,18], even if there is only few HCV RNA available.

To this end, in simple models of the type introduced in our former paper [34], the transport of HCV RNA into the geometrically defined MW regions is possible already before the MW has grown, i.e., before enough web proteins have accumulated within the corresponding web region subdomain. (These geometrically defined MW region subdomains are based upon the reconstructions of the dsRNA marked regions of the immunostained cell data.)

#### Appendix D.1.2. One Component—One Concentration

The concept used in our former work [34] was to introduce spatial resolution into computational virology. We distinguished the role of each component (HCV RNA, web protein, and host factor) only by means of its spatial location. However, this was a very first approach. Our previous model was unable to capture the biological evidence according to which a substance that is, e.g., translated, cannot be simultaneously reproduced. The source for this limitation can be viewed, for example, in Equations (2a) and (2b), where the HCV RNA concentration *R* is simultaneously produced (see the reaction term in Equation (2a)) in the subdomains W∪R and translated (see the term $r_{2}R$ in Equation (2b)) in a part of the same subdomains, namely R⊂(W∪R).

### Appendix D.2. Properties of the New Model to Heal Former Restrictions

Our new model heals various of the afore-described limitations of the former model. In Appendix C, we discuss the advantages of the new model in a detailed technical manner. Here, we analyze the reasons of the progress induced by the new model.

#### Appendix D.2.1. Backbone One—Different Aggregate States for the HCV RNA Cycle Agents

The different agents (HCV RNA and NSP) appear in different versions rather then one state described by one equation for each of the components (used before [34]). Different versions of the components mean that different modes of action of a component are respectively described by means of different concentrations. The introduction of intermediate aggregate states allowed for quasi-realistic descriptions of the single events. Different aggregate states of the different agents are realized as different concentrations. These different concentrations correspond to the different aggregate states/modes in which the agent may appear/act. The components are specified concerning their modes of action and their chemical configuration, which are not necessarily concerning the spatial location. In summary, by now, we do not only model each component by one state, but also the aggregate states of the respective components.

In particular, the uncertainty concerning the action of the HCV RNA was healed in this study by means of the introduction of different aggregate states for the HCV RNA. Oppositely to our former model [34], we may now always distinguish in which aggregate state/chemical bound state the HCV RNA appears. For example, HCV RNA that is ribosomal bound is modeled by means of another concentration as that HCV RNA that gets replicated. The different states allow for a realistic description of the single components.

In detail, only free HCV RNA may diffuse (either inside the MW regions or away from the MW regions at the ER surface), but it cannot translate polyprotein as long as it does not get captured by ribosomes, i.e., as long as it does not change its aggregate state in a clearly defined manner (Equations (5f) and (5a)). Moreover, free HCV RNA cannot be reproduced, i.e., polymerize new HCV RNA. The HCV RNA, once bound at the ribosomes, may neither diffuse away outside from the ribosomal region nor be replicated. Only ribosomal bound HCV RNA can translate viral polyproteins. However, the ribosomal bound HCV RNA may dissolute from the ribosomes to get bound together with web proteins (in a defined relation of HCV RNA to web protein number) forming the RC. However, the RC bound HCV RNA is no longer bound at the ribosomes, hence it can no longer translate polyprotein. The RC diffuses into the MW regions. Only the MW located RC may polymerize new (freely diffusing) HCV RNA.

Strange situations of that kind that HCV RNA may get translated while it diffuses away and is getting copied at the same time are no longer possible. Transitions between the different aggregate states happen by means of clearly defined reaction terms, which cause, for example, that the free HCV RNA gets “captured” by ribosomes (Equation (5a)).

Hence, the combination of intermediate aggregate states for the contributing agents allows very precise descriptions, for example for the HCV RNA reproduction.

#### Appendix D.2.2. Backbone Two—Population Dynamics

We introduced elements of population dynamics for the structure of reaction and also diffusion coefficient terms. This indicates that, for the limiting case of small concentrations, processes effectively may not take place. Only once there is enough concentration available, the process (reaction or diffusion) is “switched on”. In particular, population dynamics no longer permits artifacts, as reported in Section D.1.1.

We mention that, by setting some of the coefficients $F_{i}$, for i=0,…,11, in Equations (5a)–(5g) and (6a)–(6c), equal to zero, one can easily re-obtain a model in which some reaction terms and diffusion coefficients are linear or constant with respect to the concentrations of the considered substances. Therefore, our new population dynamics model inherits even the simple linear case as limit, which enables the choice of a simpler model within the given framework.

Nevertheless, we use population dynamics functions for reaction parameters and also for diffusion coefficients of our model. This allows for local switching-on and switching-off of processes in a way that depends upon the local concentrations. The population dynamics inspired reaction coefficients allow for special process structures. Reactions only may start or hold on and take place efficiently once a substantial concentration of the corresponding agent is reached, or as long as a substantial concentration of an agent is still present. This property ensures that a reaction may start or continue only if a substantial concentration is reached.

The feature of population dynamics for switching-on and switching-off processes depending on the concentrations is important for the construction of the RC and for the translation of polyprotein by ribosomal bound HCV RNA: One HCV RNA always is bound chemically (Equation (5a)) to a defined amount of web proteins (Equation (5d)) and forms the RC (Equation (5e)). The property of RC construction of our new model is much more realistic and biologically sound then multilinear approaches. In the new model, only if enough web protein is available, the RC can be constructed, while the RC may not be constructed as long as only few webs are available. This property reflects the need to have a substantial amount of web proteins to reproduce the HCV RNA. In addition, e.g., since the relation of length of HCV RNA to NSP size is not infinite, infinitely small NSPs might cause a linear increase of HCV RNA reproduction proportional to NSP number, however also the NSPs have a non-negligible size compared to the HCV RNA size.

HCV RNA polymerization breaks down once the host factor is reduced substantially (cf. Equation (5f)). This property causes that either a realistic amount of HCV RNA gets synthesized, or the polymerization stops effectively. This property causes that, per newly synthesized HCV RNA, the amount of consumed host factor is always nearly the same (Equation (5g)). Hence, the host factor consumption for the construction of one new HCV RNA no longer depends on the (locally or overall) available HCV RNA and web protein concentration, but only upon the amount of HCV RNA synthesized which seems to be biologically more sound. The host factor acts as some sort of catalyst for theproduction of polyprotein and RC (see Equations (5b) and (5e), and, for the case of the binding of the free HCV RNA to the ribosomes, Equations (5a) and (5f), respectively). If we set some $F_{i}$ equal to zero in Equations (5a)–(5g) for (a) certain *i*(s), then the corresponding catalyst properties vanish again, since the concentrations in numerator and denominator cancel each other and hence the population dynamics property vanishes at the corresponding parts.

The afore-discussed examples mimic quite well the realistic cases—polyprotein translation may only take place if there is really at least one HCV RNA bound at the ribosomes, and RC only may be constructed once there are a lot of web proteins available.

The population dynamics form of the reaction in Equation (5b) causes that a relatively clearly defined amount of HCV RNA concentration has to be bound at the ribosomal surface before the translation may start. (This property could be used, for example, to compute a realistic value for the population dynamics coefficients $F_{i}$ that appear in Equation (5b). For example, one could determine $F_{0}$ based upon this principle. Thus far, however, we did not consider this question in detail, since the scope of this study was to introduce the populations dynamics principles into computational virology at an intracellular level in general. More detailed questions have to remain for future work.) In the case of small concentrations of HCV RNA, the probability is small that a HCV RNA is attached to a ribosomal region. Then, translation is strongly suppressed.

Population dynamics are also not used thus far within the state-of-the-art-models that use multilinearity, to the best of our knowledge (cf. [34] and references therein).

#### Appendix D.2.3. Backbone Three—Spatial Resolution on Realistic Geometries

Of course, the major backbone of our framework is the full 3D spatial resolution upon realistic reconstructed geometric structures, namely the ER surface and the MW regions (cf. Section 2.3).

To this end, this framework allows for realistic simulations even with simple models [34] and for very realistic simulations in combination with advanced models, as presented within this study.

## Appendix E. Surface and Volume Models

There is biologic evidence that the ER surface plays an important role on the movement of viral proteins. In particular, the NSPs anchor to the surface of the ER directly after their cleavage from the viral polyprotein. For this reason, and because the ratio volume-to-surface of the MW regions is negligibly small, we assumed that the diffusion process of the viral proteins can be reliably modeled as if it took place only on the ER surface and on the surface of the MW regions. This is, however, a first approximation since the viral proteins may in fact move both on the ER surface and *within* the web regions. Moreover, since there is no detailed experimental information available referring to the HCV RNA movement properties, in our approach, we restricted also the HCV RNA movement to the ER surface.

### Appendix E.1. Mixing Surface and Volume Models

The vPDE model developed in our former study [34] was built within the complete volume of the cell by means of a volume grid approach, but restricted to very simple PDE descriptions. Therefore, in model extensions, we intend to allow host factor initial value and diffusion within the complete volume, but with NSP movement restricted to the ER surface. This extension seems to be biologically sound (since there is no reason to assume host factor movement is limited to any surface). Such a property will have impact upon the quantitative aspect of data, but not so much to qualitative aspects. In any case, the important qualitative property of the host factor transport influence (as demonstrated already in our former more simple model [34]) likely will remain valid also in such contexts. Such an influence of the interplay of form and function can only be detected within spatially resolved models.

Clearly, the surface model presented in this manuscript is unable to account for the possible onset of high volume concentration gradients that may be established between neighbouring folds of the ER surface. However, the surface model features major aspects that cannot be resolved in the pure volume model. One of those, for instance, is the motion of the NSPs, which is in fact restricted to occur only on the ER surface. Such a surface motion, indeed, cannot be correctly modeled in a model that is evaluated within a pure volume grid. On the other hand, there could be phenomena that take place both on the two-dimensional ER surface and in three-dimensional regions, as could be the case for the diffusion of the host factor in the cytoplasm among the folds of the ER, and are thus not sufficiently well described by our surface model. For these reasons, it could be very interesting to combine the surface and the volume models, and capture the characteristic features of each of those. In undertaking this task, one should consider, however, that some processes concerning the viral replication dynamics are not yet sufficiently understood. For example, this is the case of the motion of the RNA, for which—to the best of our knowledge—it has not been experimentally observed whether it occurs in the cytoplasm or only on the ER surface. To improve the comprehension of such processes, it could be an important middle term goal to couple the surface (i.e., effective two-dimensional 3D embedded manifold) model with the pure volume (i.e., fully three-dimensional) model.

Before undertaking this task, it should be recalled that the current surface model is meant to be qualitative: Restricting the considered effects only to the ER surface is satisfactory only as a first approximation. In this respect, merging the surface and volume models may bring not much new information from the qualitative point of view, but it may produce quantitative differences. Such merging, however, is rather challenging from the technical point of view. Since our scope is the introduction of a spatial resolution and of a detailed description of the dynamics of the single components taking part of the viral replication cycle, we limit ourselves for the time being to the presented surface model and we would investigate in the future the possibility of merging the surface model with the volume model.

## Appendix F. Refinement Studies

We investigate the stability of the concentrations in integrated form and along the afore indicated paths.

We observe good grid convergency behavior of all components for both data types. The changes that appear decrease at each increase of the grid level. This is in agreement with the need for grid convergency. We take these results as strong hint that our computations are also well-founded with respect to the numerical results, as well as as a strong hint that the equations are well-defined. (Indeed, elaborated proofs for well-definedness of highly nonlinear coupled PDE systems in 3D are a complex task. To the best of our knowledge, until now there is still a lack of such proofs even for the general case of Navier–Stokes equations when evaluated within three dimensions.) Anyhow, even though we did not give a technical proof for the existence and uniqueness of solutions of our PDE system, it is necessary to have at least strong hints for mathematical well-posedness. We choose grid convergency behavior of our results as an important part of our evidence based “proof”. We leave an extended strict mathematical proof to further studies, which we intend to do in the future.

### Appendix F.1. Integrals of Concentrations Under Grid Refinement

Figure A1 (viral protein components) and Figure A2 (HCV RNA containing species and host factor) depict the refinement behavior of the integrated concentrations over the complete computational domain. We show absolute values and relative differences for levels 0, 1, 2, 3 and 4.

### Appendix F.2. Concentration Profiles along 1D Trajectories Under Grid Refinement

In Figure A3, Figure A4, Figure A5, Figure A6 and Figure A7 we depict the grid refinement behavior and the relative differences of the concentrations of the components which appear at the respective pathways of the short paths surrounding a web.

Figure A8 depicts the grid convergency behavior of the concentrations of free HCV RNA and host factor along the long path for t=3 s.

In the case of long and short paths, we report the absolute values for and the relative changes among levels 1, 2, 3 and 4.

## Appendix G. Mathematical Operators of Derivation, Tangential Part, and Biological Context

### Appendix G.1. Mathematical Definitions of div_T_ and *∇*_T_ and Biological Meanings

For the sake of completeness, we recall here the definitions of gradient and divergence operators, and we discuss what they represent in the biological context presented in our work.

Let Ω⊂S be an open set of the three-dimensional Euclidean space, S, and let f:Ω→R be a scalar field defined over Ω. If all the partial derivatives of *f* exist everywhere in Ω, the gradient (“grad” or ∇) of *f* at a given point x∈Ω is defined as [65,66].

(A1) $$\begin{array}{cc} \mathrm{grad}f(x)\equiv \nabla f(x) & =\frac{\partial f}{\partial x_{1}}\left(x\right)e_{1}+\frac{\partial f}{\partial x_{2}}\left(x\right)e_{2}+\frac{\partial f}{\partial x_{3}}\left(x\right)e_{3}\\ & =\sum_{k=1}^{3}\frac{\partial f}{\partial x_{k}}\left(x\right)e_{k}, \end{array}$$

where ${\left\{e_{k}\right\}}_{k=1}^{3}$ is a Cartesian triad of basis vectors, and the triple of real numbers $\left(x_{1},x_{2},x_{3}\right)$ denotes the coordinates of x∈Ω.

Moreover, given a vector field u over $\Omega \subset R^{3}$, and adopting the decomposition

(A2) $$\begin{array}{c} u=u_{1}e_{1}+u_{2}e_{2}+u_{3}e_{3}, \end{array}$$

the triple of real-valued functions $\left(u_{1},u_{2},u_{3}\right)$ represents the components of u in the Cartesian vector basis ${\left\{e_{k}\right\}}_{k=1}^{3}$. If each function $u_{k}$, for k=1,2,3, is differentiable, the “gradient of u” at x∈Ω is defined by

(A3) $$\begin{array}{c} \mathrm{grad}\;u\left(x\right)\equiv \nabla u\left(x\right)=\sum_{m,n=1}^{3}\frac{\partial u_{m}}{\partial x_{n}}\left(x\right)e_{m}⊗e_{n}, \end{array}$$

where the symbol “⊗” denotes the dyadic product between vectors. We recall that, for any pair of vectors v and w, the dyadic product v⊗w is the second-order tensor with components ${\left[v⊗w\right]}_{mn}=v_{m}w_{n}$. Finally, the divergence of u is obtained by contracting the two indices of gradu [65], i.e.,

(A4) $$\begin{array}{c} \mathrm{div}\;u\left(x\right)=\frac{\partial u_{1}}{\partial x_{1}}\left(x\right)+\frac{\partial u_{2}}{\partial x_{2}}\left(x\right)+\frac{\partial u_{3}}{\partial x_{3}}\left(x\right)=\sum_{k=1}^{3}\frac{\partial u_{k}}{\partial x_{k}}\left(x\right). \end{array}$$

Note that, if gradu(x) exists, divu(x) can be computed as the trace of the matrix representation of gradu(x).

In our work, we make frequent use of the gradient and divergence operators, since both operators feature in the diffusion–reaction equations on which our model is based. In fact, each of such equations represents the mass balance law for a given species of the model, and can be written as

(A5) $$\begin{array}{c} \partial_{t}c=-\mathrm{div}q+r, \end{array}$$

where *c* is the concentration of the considered species, q is the mass flux vector describing the motion of the species, and *r* is the corresponding source or sink term.

Equation (A5) is the local form of the mass balance law of mass, and descends from the integral form

(A6) $$\begin{array}{c} \frac{d}{dt}\int_{P}c\;dv=-\int_{\partial P}q\cdot n\;da+\int_{P}r\;dv. \end{array}$$

In Equation (A6), P⊆Ω is a generic part of Ω, ∂P is its boundary of *P*, n is the field of unit vectors normal to ∂P, and dv and da denote volume and surface measures, respectively. The physical (and biological) meaning of Equation (A6) is that the mass of the species contained in *P* with the concentration *c* varies is time in response to the flux of the species through ∂P, and to its production or depletion in *P*. The flux of the species through ∂P is given by the component of q along n, obtained by the scalar product q·n. By prolonging q to the interior of *P*, and assuming differentiability, Gauss’ Theorem can be invoked to recast Equation (A6) as

(A7) $$\begin{array}{cc} \frac{d}{dt}\int_{P}c\;dv= & -\int_{P}\mathrm{div}q\;dv+\int_{P}r\;dv\\ & =\int_{P}\left[-\mathrm{div}q+r\right]dv. \end{array}$$

Assuming that the part *P* of Ω is fixed, the time derivative of the volume integral of *c* can be rewritten as the volume integral of the partial time derivative of *c*, thereby yielding

(A8) $$\begin{array}{c} \int_{P}\partial_{t}c\;dv=\int_{P}\left[-\mathrm{div}q+r\right]dv. \end{array}$$

Furthermore, requiring that Equation (A8) applies for *any* choice of *P* allows rewriting Equation (A8) as reported in Equation (A5).

In summary, the use of the divergence of the mass flux vector, i.e., divq, allows transforming the surface integral of q·n featuring in Equation (A6) into the volume integral of divq featuring in Equations (A7) and (A8).

Equation (A5), or the integral form in Equation (A6), involves two scalar unknowns, i.e., *c* and *r*, and the vectorial unknown q, which introduces the triple of unknown scalar components $\left(q_{1},q_{2},q_{3}\right)$. Hence, to close the mathematical problem associated with Equation (A5), it is necessary to provide constitutive relationships connecting q and *r* with *c*, or prescribing them from the outset. Here, we focus on the relationship that, on physical grounds, has to be supplied in order to relate q to *c*.

If we assume that the motion of the considered species occurs by diffusion, we need to describe the spontaneous and irreversible process by which the motion of the species is driven by the spatial non-uniformity of its distribution. In other words, if one prepares an experiment in which the concentration of the species, *c*, varies in space, a motion of the species can be observed that tends to restore a space-independent concentration. For this reason, and since the descriptor of the spatial variability of *c* is the concentration gradient, ∇c, we identify ∇c as the trigger of the species’ motion. If the medium in which the species evolves is isotropic, the easiest way to relate q to ∇c is to assume a linear relationship of the type q=−D∇c, where *D* is referred to as coefficient of diffusion. We remark that many other laws of this kind are to be found in physics, biology and engineering: Fick’s law of molecular diffusion, Fourier’s law of heat conduction, Darcy’s law of fluid filtration through porous media, and Ohm’s law of electric conduction are perhaps the most prominent examples. The expression of q used in our work, i.e., q=−D∇c, is in fact a particular case of Fick’s law of diffusion. By substituting it into Equation (A5), we find

(A9) $$\begin{array}{c} \partial_{t}c=\mathrm{div}\left(D\nabla c\right)+r. \end{array}$$

In Equation (A9), the reaction term, *r*, can be either prescribed from the outset or related to *c* through a constitutive law of the type $r=\hat{r}\left(c\right)$.

In our work, to describe the diffusion of a given substance on the (open) surfaces that represent our computational domains, we use the tangential gradient and the tangential divergence operators. For the scalar field *c*, the tangential gradient is denoted by $\nabla_{T}c$ and is defined as

(A10) $$\begin{array}{c} \nabla_{T}c=\left[\nabla c\right]P, \end{array}$$

where P=I−n⊗n is said to the *projection operator*, I is the identity tensor, and n is field of unit vectors normal to the surface S over which the diffusion takes place. For Equation (A10) to make sense, *c* has to be restricted to S. Hence, *c* has to be understood as $c_{|S}$ in Equation (A10). Accordingly, the tangential mass flux vector associated with $c_{|S}$ is given by $q^{\sigma }=-D\nabla_{T}c$.

To write the mass balance law on S, one has to take the tangential divergence of the mass flux vector. In general, for a vector field ψ defined on S, but that is not necessarily tangent to S, the tangential divergence is defined as [67]

(A11) $$\begin{array}{c} {\mathrm{div}}_{T}\psi :=\mathrm{tr}\left(P\nabla_{T}\psi \right)={\mathrm{div}}_{T}\psi^{\sigma }-2\kappa \psi_{n}, \end{array}$$

where tr(•) is the trace operator, $\psi^{\sigma }=P\psi$ is the projection of ψ onto S, κ is the mean curvature of S, and $\psi_{n}$ is the component of ψ normal to S. We emphasize that, for a tangential vector field, as is the case for $q^{\sigma }$, it holds that $Pq^{\sigma }=q^{\sigma }$, and Equation (A11) becomes independent of the mean curvature, thereby reducing to

(A12) $$\begin{array}{c} {\mathrm{div}}_{T}q^{\sigma }=-{\mathrm{div}}_{T}\left(D\nabla_{T}c\right). \end{array}$$

Accordingly, the diffusion–reaction equation (Equation (A9)) becomes

(A13) $$\begin{array}{c} \partial_{t}c={\mathrm{div}}_{T}\left(D\nabla_{T}c\right)+r, \end{array}$$

and is the local form of the integral equation

(A14) $$\begin{array}{c} \frac{d}{dt}\int_{A}cda=\int_{\partial A}\left(-D\nabla_{T}c\right)\cdot \nu \;ds+\int_{A}rda, \end{array}$$

where A⊆S is a subsurface of S, ∂A is a closed and regular line, contained in S and defining the boundary of A, ν is a field of unit vectors normal to ∂A and tangent to A, and ds is a line measure.

The biological meaning of the use of the tangential space operators is that they allow for restricting movement of components to a 2D surface embedded within 3D, e.g., to restrict the movement of biological agents such as proteins to the ER surface.

### Appendix G.2. Biological Motivations to Use the Operators div_T_ and *∇*_T_

In summary, the divergence of the mass flux vector, divq, descends from Gauss’ Theorem, and permits expressing the species’ mass balance law in local form. However, as shown in Equation (A6), one can dispense with divq, if the mass balance law is formulated in integral form. This occurs, for instance, if a finite volume method is applied to solve Equation (A6), as is the case in our simulations. Indeed, when the Finite Element Method is employed, the part *P* of Ω is referred to as *finite volume*, and the quantity q·n is evaluated only at selected points of ∂P, which are said to be *integration points*. Moreover, upon substituting q with its explicit expression, Equation (A6) becomes

(A15) $$\begin{array}{c} \frac{d}{dt}\int_{P}c\;dv=\int_{\partial P}D\nabla c\cdot n\;da+\int_{P}r\;dv. \end{array}$$

The presence of the concentration gradient ∇c in Equation (A15) is an essential feature of our model, which aims to *resolve explicitly* the spatial variability of *c*. Indeed, if the first term on the right-hand-side of Equation (A15) were suppressed from the outset, our model would boil down to a standard description of virus dynamics, entirely based on ordinary differential equations. Models of this kind supply indications about the time evolution of averaged values of the concentration of a given species, but they are not designed to keep track of the spatial distribution of such a concentration. In this respect, the biological motivation for using ∇c in our model is the need for resolving in space the variability of *c*. The knowledge extracted by such information is expected to improve our understanding of the processes underlying the virus dynamics in the cells.

Within our model approach, we use div${}_{T}$ and $\nabla_{T}$ rather then the normal div and grad operator. This means that we use only that part of div and grad which belongs to the tangential space of the 2D ER manifold as embedded within the full 3D space.

This technique is motivated by the fact that the nonstructural viral proteins anchor to the ER surface directly after their cleavage from the polyprotein. Their movement is restricted to the ER surface. Hence, our surface PDE approach mimics the restriction of NSP movement to the ER surface. For reasons explained in the main part, we restrict also the movement of the other components to the ER surface. However, this restriction is preliminary and we intend to overcome this limitation within forthcoming model simulations.

## Appendix H. Surface Reconstruction Process with NeuRA2

The reconstructions of the ER surfaces were performed with the aid of NeuRA2 [35,36,46,47]. The single steps read:

- Deblurring of the separate channels of ER surface and MWs (done with Huygens [68]),
- For each channel, i.e., ER and MWs, separate surface reconstruction (performed with NeuRA2.3 [36,46,47]):
  – shaping the ER surfaces and the web regions with inertia momentum based anisotropic filtering [46,69], cf. also Appendix H.1, i.e., directly below
  – either Hysteresis or Otsu [70] segmentation,
  – surface reconstruction of ER and MW regions with the marching cubes algorithm
- Merging and improvement of the two different subdomain types (ER surface and MW regions) within ProMesh [71].

### Appendix H.1. The Inertia Momentum-Based Anisotropic Filter

The inertia momentum-based anisotropic diffusion filter reduces the noise while it conserves the size of the structures in microscopy pictures. It has a central role in our reconstruction process, as described in the basic literature [46,69]. The basic idea is that the intensity of the pixels is interpreted as the distribution of some physical quantity undergoing anisotropic diffusion. The diffusion tensor of this fictitious diffusion process is computed by evaluating the movement of inertia tensor associated with the pixel intensity. Since the algorithm asks for the solution of huge linear equation systems, which have in principle simple structures, it was implemented for GPGPU environments, which allow for a very time efficient application [47].

## Appendix I. Analysis of Population Dynamics Coefficient Structure

Our form of population dynamics depends on the mathematical form of the powers used in numerator and denominator. The standard form of a population dynamics-based process depending on the concentration of an ingredients reads

(A16) $$f\left(x\right)=\frac{x^{a}}{x^{a}+b}.$$

Figure A9 shows examples of the choice of powers a=1,4,6 and different values of the regulator *b*.

The thresholds and the sharpness of the transitions between different process stages depend on the factors and powers of the respective population dynamics inspired reaction and diffusion coefficients (e.g., see Figure A9).

The standard form of population dynamics of the form x/(a+x) causes slow growth processes of factors when concentration augments, however does not allow for mimicking processes that start only if a sufficient high concentration is accessible. Therefore, different ways of powers of the form $x^{i}/\left(b+x^{i}\right)$ were examined by us. As shown in Figure A9, we compared different scenarios of multiplication functions, which led us to the decision to use power 4 factors instead of factor 1 in some cases, since the starting points and the breakdown points are much clearer, indicating similarities to the frontier between single particle and continuum models. Hence, we found that factor 4 is very useful in mimicking processes that start only if a substantial concentration is available, but in this case nearly instantaneously.
